# Probing the atomic dynamics of ultrafast melting with femtosecond electron diffraction

**DOI:** 10.1038/s41467-026-75970-1

**Published:** 2026-08-06

**Authors:** M. Z. Mo, M. B. Maigler, T. Held, B. K. Ofori-Okai, A. Bergermann, Z. Chen, R. K. Li, X. Shen, K. Sokolowski-Tinten, R. Redmer, X. J. Wang, J. Schein, D. O. Gericke, B. Rethfeld, S. H. Glenzer

**Affiliations:** 1 SLAC National Accelerator Laboratory, Menlo Park, California USA; 2 Bundeswehr University Munich, Neubiberg, Germany; 3 Department of Physics and Research Center OPTIMAS, RPTU University Kaiserslautern-Landau, Kaiserslautern, Germany; 4 Institut für Physik, Universität Rostock, Rostock, Germany; 5 Faculty of Physics and Centre for Nanointegration Duisburg-Essen (CENDIE), University of Duisburg-Essen, Duisburg, Germany; 6 Helmholtz-Zentrum Dresden - Rossendorf, HEDI - Institute of High Energy Density Physics, Rostock, Germany; 7 Faculty of Physics, University of Duisburg-Essen & Research Center Chemical Sciences and Sustainability, Duisburg, Germany; 8 Department of Physics, TU Dortmund University, Dortmund, Germany; 9 Centre for Fusion, Space and Astrophysics, Department of Physics, University of Warwick, Coventry, United Kingdom

**Keywords:** Nanoscale materials, Nanoscale materials

## Abstract

Melting is an every-day phase transition that is determined by thermodynamic parameters like temperature and pressure. In contrast, ultra-fast melting is governed by the microscopic response to a rapid energy input and, thus, can reveal the strength and dynamics of atomic bonds as well as the energy flow rate to the lattice. Accurately describing these processes remains challenging and requires detailed insights into transient states encountered. Here, we present data from femtosecond electron diffraction measurements that capture the structural evolution of copper during the ultrafast solid-to-liquid phase transformations. At absorbed energy densities 2-4 times the melting threshold, melting begins at the surface slightly below the nominal melting point followed by rapid homogeneous melting throughout the volume. Molecular dynamics simulations reproduce these observations and reveal a weak electron-lattice energy transfer rate for the given experimental conditions. Both simulations and experiments show no indications of rapid lattice collapse when its temperature surpasses proposed limits of superheating, providing evidence that the inherent dynamics limits the speed of disordering in ultrafast melting of metals.

## Abstract (web summary)

Despite recent experiments, an accurate and unifying description of laser-induced ultrafast melting is still lacking. Here, the authors apply ultrafast electron diffraction measurements to probe picosecond-scale structural evolution of copper during the solid-to-liquid phase transition, providing critical insight into its transient states and melting pathway.

## Introduction

Rapid energy deposition into a material drives a cascade of kinetic and structural processes^1,2^. The energy source, typically a short-pulse laser, usually couples almost entirely to the electrons, driving them into a state far from equilibrium. For metals, the subsequent relaxation to Fermi-distributed electrons occurs on femtosecond time scales^3–5^. Afterward, the material can often be modeled as a two-temperature system with hot electrons and a cold lattice^6,7^, but the electronic band occupation and nuclei positions may still not be in equilibrium^8,9^. The energy transfer from the electrons to the lattice is the slowest kinetic process and finally heats the lattice via electron-phonon coupling. Once the lattice reaches its melting temperature, one expects a phase transition, but nucleation kinetics competes with further lattice heating, and the loss of lattice structure may be considerably delayed^10–16^.

Understanding the chain of processes that leads to ultrafast melting of metals and, in particular, the time scales for all relaxation stages is crucial for a wide range of applications, including laser micromachining^17^, studies of planetary interiors^18,19^, inertial fusion experiments^20,21^, laser-based colloid synthesis^22^, and material modifications^23^. Each of these applications would benefit greatly from thorough modeling capabilities that can reduce requirements for experimental tests, enhance analysis, or guide design. However, quantitative modeling requires knowledge of all energy transfer pathways and has to take into account the complex interplay of the different relaxation processes.

Recent results for copper^24–28^ show that models for the dynamic response after rapid energy deposition still lack predictive power. Indeed, these experimental data are highly controversial and show significant disagreements with standard modeling and theory^29–31^. One major source of uncertainty is the indirect determination of the temperature and time of the phase transition. Here, we address this limitation by combining ultrafast electron diffraction experiments with atomistic simulations to investigate structural dynamics on the picosecond time scale where melting occurs. Ultrafast electron diffraction^13^ allows direct access to the dynamics of the phase transition on the atomic scale. Our data show clear signatures for structural changes, and the analysis of the transient structure allows for the determination of the lattice temperature via the Debye-Waller factor. The latter gives access to the rate of electron-phonon energy transfer. The experimental data are supplemented by Molecular Dynamics (MD) simulations for the copper nuclei coupled to heated and equilibrated electrons^32^. By using a highly optimized interatomic potential and an electron-phonon coupling strength validated by our experiments, we obtain good agreement with the measured melting kinetics, including the onset, duration and completion of melting.

## Results

### Ultrafast electron diffraction

**Fig. 1:**
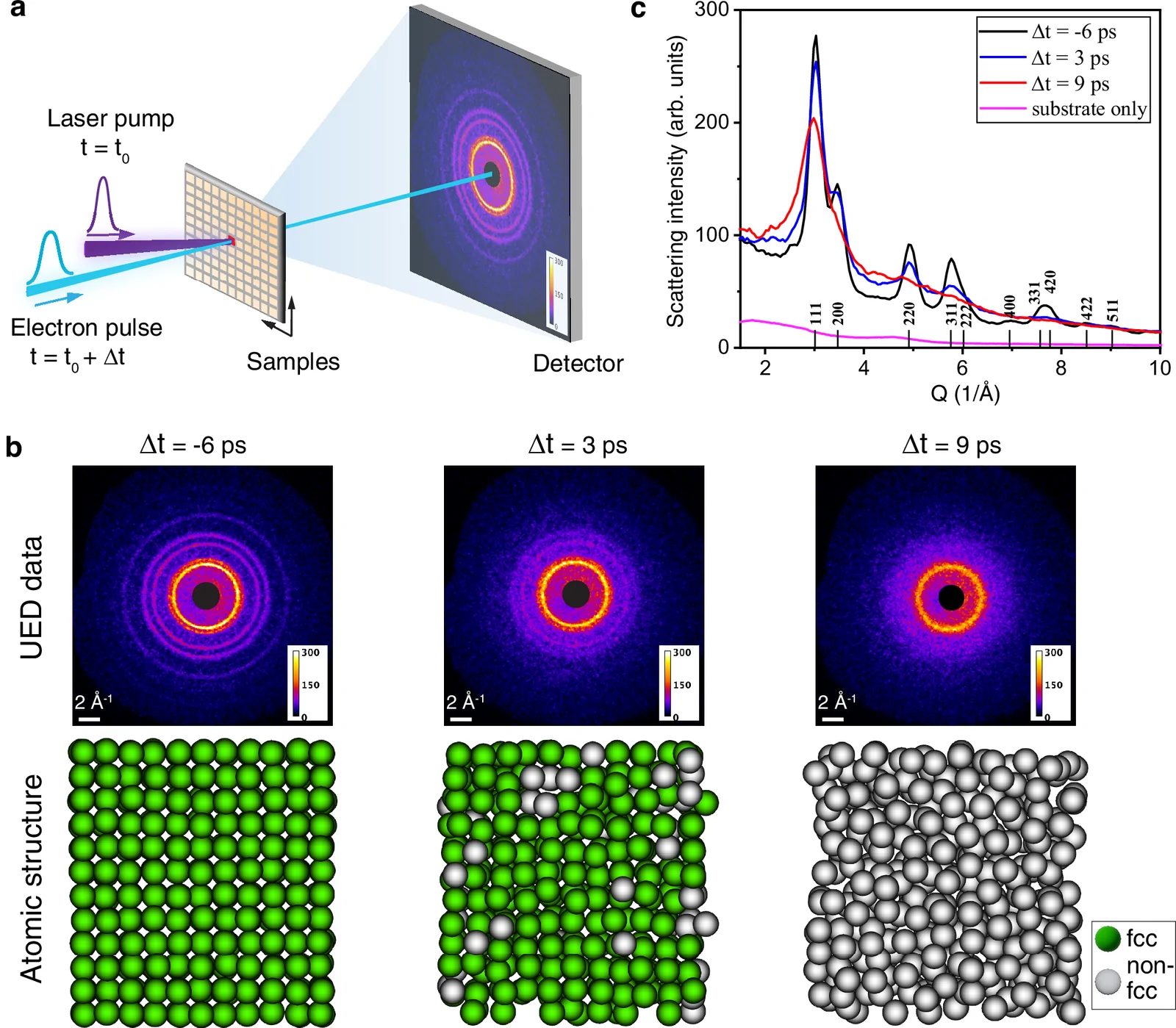
Electron diffraction study of ultrafast melting of copper. **a** Schematics of the experimental setup. The copper targets were pumped by intense 130 fs, 400 nm laser pulses and the induced solid-liquid phase transitions were probed with time-resolved electron diffraction using 350 fs pulses of 3.2 MeV electrons. **b** Top panel: sequence of diffraction patterns obtained at selected times over the solid-liquid phase transition obtained for an energy density of 2.55 MJ kg^−1^. Bottom panel: snapshots of atomic configuration in a small subvolume as obtained by MD simulations to illustrate the transient atomic structures at corresponding time delays. Green spheres are atoms in the fcc phase, while gray spheres are non-fcc (liquid) atoms. **c** Radially averaged scattering intensity of the diffraction patterns shown in (**b**). The vertical black lines indicate the positions of diffraction peaks for fcc copper. For comparison, the intensity for only the unpumped Si_3_N_4_ substrate was included.

Figure 1 a shows the schematic diagram of our experimental setup. We used 40 nm-thick polycrystalline copper films deposited on 30 nm-thick, free-standing, amorphous Si_3_N_4_ membranes as targets for our ultrafast melting study. We excited these targets from the copper side by 130 fs long [full width at half maximum (FWHM)] laser pulses with a wavelength of 400 nm and flat-top-like intensity profiles of ∼ 400 *μ*m diameters. We expect uniform heating in the longitudinal direction because of the ballistic energy transport from the non-thermal electrons excited by the laser pulses, which have a mean free path of approximately 70 nm^24^. The incident laser fluence during the measurement was set between 105 mJ cm^−2^ and 213 mJ cm^−2^, corresponding to absorbed energy densities ranging from *ϵ* = 1.26 ± 0.19 MJ kg^−1^ to *ϵ* = 2.55 ± 0.38 MJ kg^−1^. Thus, the energy densities in the target exceed the threshold of complete melting, that is ∼ 0.62 MJ kg^−1^ (see “Methods”), by a factor of two to four.

To investigate the dynamic structural changes during the melting processes, we carried out ultrafast time-resolved electron diffraction measurements in a normal incidence transmission geometry, utilizing bunches of 3.2 MeV (kinetic energy) electrons with bunch charges of ∼ 20 fC, and pulse durations of ∼ 350 fs (FWHM). We focused these relativistic electrons to diameters of ∼ 120 μm on the targets. The scattered electrons were recorded by a phosphor-based detector located 3.2 m downstream, providing a maximum momentum transfer *Q* of ∼ 10 Å^-1^. Here, *Q* is defined as $Q=4\pi \sin \theta /\lambda$ with *θ* and *λ* representing the scattering angle and the de Broglie wavelength of the electrons, respectively. At each pump fluence, we scanned pump-probe time delays (*t*) over several tens of picoseconds with 0.5 ps time steps near time zero and larger intervals thereafter. At each time delay, we acquired and averaged a total of eight pump-probe events, resulting in diffraction patterns with excellent signal-to-noise ratios.

In Fig. 1b, we present a series of electron diffraction patterns taken before, during and after the solid-liquid phase transition for *ϵ* = 2.55 MJ kg^−1^. At *t* = − 6 ps, far before the laser arrival, the diffraction pattern shows distinct Debye-Scherrer rings that are characteristic of the face-centered cubic (fcc) structure of polycrystalline copper. The radially averaged intensity lineout shown in Fig. 1c reveals a substantial background present below the diffraction peaks. This background predominantly arises from various scattering phenomena inside the copper film, including multiple elastic scattering and inelastic scattering events such as thermal diffuse scattering^33^. In the case of a medium atomic-number material like copper, thermal diffuse scattering is expected to dominate the background, given the relatively large elastic mean free path (∼ 65 nm) of MeV electrons in copper.

At *t* = 3 ps, the diffraction pattern displays a considerable qualitative change, marked by the vanishing of diffraction rings in the high-*Q* region and a reduction in the intensity of rings at low *Q*. By contrast, the overall background signal is greatly enhanced. These observed changes are further illustrated by the scattering intensity lineout shown in Fig. 1c. It’s worth noting that the diffraction pattern lacks a distinct feature of the liquid structure factor, which would otherwise broaden the width of the (111) peak and downshift its position in *Q*, as shown by the intensity lineout taken at later times. This suggests that the structure of the excited sample remains predominantly in a solid state at *t* = 3 ps.

At a much later time, i.e., *t* = 9 ps, the pattern is characterized by a broad scattering ring near the (111) peak accompanied by a highly diffusive background. This is a signature of the liquid structure factor and implies that the sample has transitioned into a completely disordered state. Thus, melting is completed at this time.

### Time-resolved electron scattering measurements

**Fig. 2:**
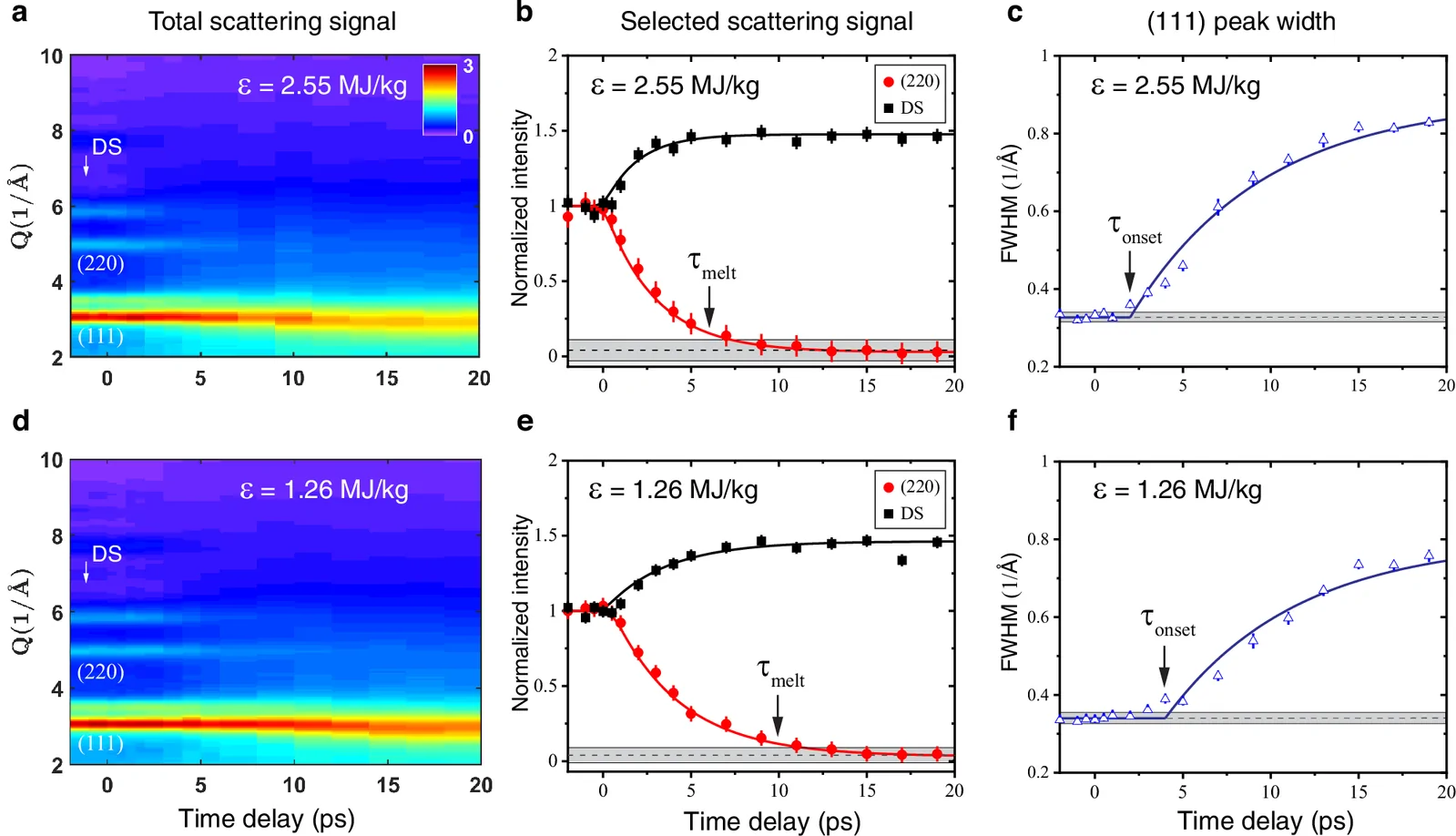
Temporal evolution of the electron scattering signal. The upper panels are results for an energy density of 2.55 MJ kg^−1^, the lower panels are for 1.26 MJ kg^−1^. **a**, **d** Time-resolved result of the total scattering signal. **b**, **e** Evolution of the intensity of the decaying (220) peak (red spheres) and the rise of diffuse scattering (DS) signal at *Q* = 6.70 Å^−1^ (black squares). Error bars represent one standard deviation (SD). The gray band represents the ground intensity level (mean value  ± 1 SD) reached at late times. The vertical arrow marks the time of complete melting τ_melt_. To account for the relatively large time intervals at late times, we define τ_melt_ as half of the time interval (1 ps) preceding the drop in (220) intensity to the liquid scattering level. **c**, **f** Time-resolved width of the (111) peak (FWHM) to monitor the signal from liquid scattering developing during the melting process. The gray band represents the initial constant level of the (111) peak width (mean value  ± 1 SD). The onset of changes in the width (τ_onset_) is marked by the vertical arrow.

To better understand the dynamics of melting and its dependence on energy density, we show the complete time history of the electron scattering signal obtained for two energy densities in Fig. 2. We find that the evolution of the total scattering signal exhibits qualitative similarities for the two excitation conditions as they both feature: (i) an intensity decay of the Laue diffraction peaks, which is evident through the declining trend of the (220) peak; (ii) an increase of diffuse scattering signal spanning over the entire *Q* range, which is, however, more visible between diffraction peaks; (iii) the rise of the liquid scattering peak, with its primary peak position slightly below the (111) peak.

To examine the first two effects, we select the signals of the (220) peak centered at *Q* = 4.92 Å^−1^ and the diffuse scattering at *Q* = 6.70 Å^−1^, and follow their time evolution. The results are shown in Fig. 2b, e. To quantify the dynamics of these transient signals, we fit the results with exponential functions defined by $1+A(1-\exp (-t/\tau ))$ with *A* being the change of amplitude at steady state and *τ* the time constant. At 2.55 MJ kg^−1^, we observe that the (220) peak decays on a time scale of 2.9 ± 0.2 ps, as comparing to 1.8 ± 0.6 ps for the rise of the diffuse scattering signal. For the lower energy density of 1.26 MJ kg^−1^, we find slower changes with time constants of 4.3 ± 0.4 ps and 3.2 ± 0.5 ps for the signals of the (220) peak and diffuse scattering, respectively. It’s crucial to recognize that under strongly driven conditions, both lattice heating and structural phase changes contribute to the observed dynamics of the total scattering signal. While the diffuse scattering shows higher sensitivity, the polycrystalline nature of the sample prevents a clear interpretation of the underlying physical mechanisms. In contrast, the decay of solid diffraction peaks allows to extract quantitative information on lattice heating and structural phase changes.

The formation of the liquid peak during the melting process cannot be analyzed in the same way as it overlaps with solid diffraction peaks, particularly in the vicinity of the (111) peak, where it is the strongest. This overlap makes it difficult to differentiate the two sources contributing to the local scattering intensity. To circumvent this issue, we instead track the width of the signal around the (111) peak, avoiding the need of decoupling the overlapping peak while still effectively characterizing the dynamics of liquid formation. This method relies on the principle that the overlapping peak width expands as crystallites decrease in size^34^ and the disorder effect intensifies^35^ during the melting process. The results are shown in Fig. 2c, f for the two energy densities applied. We observe that the onset of width broadening exhibits a delay of ∼ 2 ps and ∼ 4 ps with respect to the excitation time. We attribute this broadening to the onset of melting (*τ*_onset_), corresponding to the time required to heat the lattice close to the melting temperature. A recent time-resolved X-ray diffraction study on ultrafast melting of polycrystalline palladium thin films found that laser-induced inhomogeneous strain would contribute to the (111) peak width broadening at energy densities near the melting threshold^15^. The onset of this effect, independent of excitation strength, was observed at 3 ps after laser excitation. In our case, we cannot resolve this effect due to the limited momentum resolution of the electron scattering experiment. Besides, the clear dependence of *τ*_onset_ on excitation strength implies that laser-induced strain has a minor contribution in our case.

We find that at the onset of melting *τ*_onset_, the lattice temperature is approximately 1000 K for the stronger excitation (as described later in the text), amounting to ∼ 75% of the nominal melting temperature (*T*_melt_ = 1358 K of copper^36^). This suggests that the liquid nucleation is initiated by surface melting, which occurs on the free surface and grain boundaries at temperatures below *T*_melt_^37,38^. For the lower energy density (1.26 MJ kg^−1^), it takes longer to reach the temperature sufficient for surface melting, hence resulting in a larger *τ*_onset_. After melting has started, the width of the (111) peak can be approximated by exponential growth. A second trend may be present during the melting process and could arise from the evolving mixing of the solid diffraction peak and liquid scattering at different *Q* positions. However, for consistency with the analysis of the diffraction peak and diffuse scattering signal, we focus on the extracted time constants rather than the detailed evolution. Nonetheless, in contrast to the decay of the (220) peak, both cases here exhibit similar time constants of approximately 7.5 ± 1.0 ps, despite differences in the final equilibrated values at late delay times. As the width is connected to the rise of the liquid peak, the exponential form and the comparable time scales are indicative of homogeneous melting with similar liquid nucleation rates once the melting has started.

Our simultaneous measurement of the multiple orders of Laue diffraction peaks allows to quantify the lattice heating through the Debye-Waller factor (DWF)

1 $${{{\rm{DWF}}}}_{{{\rm{hkl}}}}(t)=\frac{{I}_{{{\rm{hkl}}}}(t)}{{I}_{{{\rm{hkl}}}}^{0}}=\exp \left[-\frac{1}{3}\Delta \langle {u}^{2}\rangle (t)\cdot {Q}_{{{\rm{hkl}}}}^{2}\right],$$

where ${I}_{{{\rm{hkl}}}}(t)/{I}_{{{\rm{hkl}}}}^{0}$ denotes the peak intensity of reflection (hkl) at time *t* normalized to its value for a cold lattice and *Q*_hkl_ is the momentum transfer of this reflection. This ratio is related to the time-dependent change of the mean square displacement of nuclei $\Delta \langle {u}^{2}\rangle (t)$ which, in turn, is connected to the lattice temperature. Our data analysis of the DWF included the (111), (200), (220) reflection peaks, and the average of the (311) and (222) reflections, which are in close proximity. To minimize the effect of shot-to-shot variations in the electron bunch charge, we apply the DWF of the (111) peak as a reference to normalize higher-order DWFs (see Supplementary Information). To additionally minimize the influence of hydrodynamic expansion and structural phase changes, we limit the DWF analysis to the initial 4 ps after laser excitation, which is half the time scale we estimate for the hydrodynamic response to become relevant (see Supplementary Information).

**Fig. 3:**
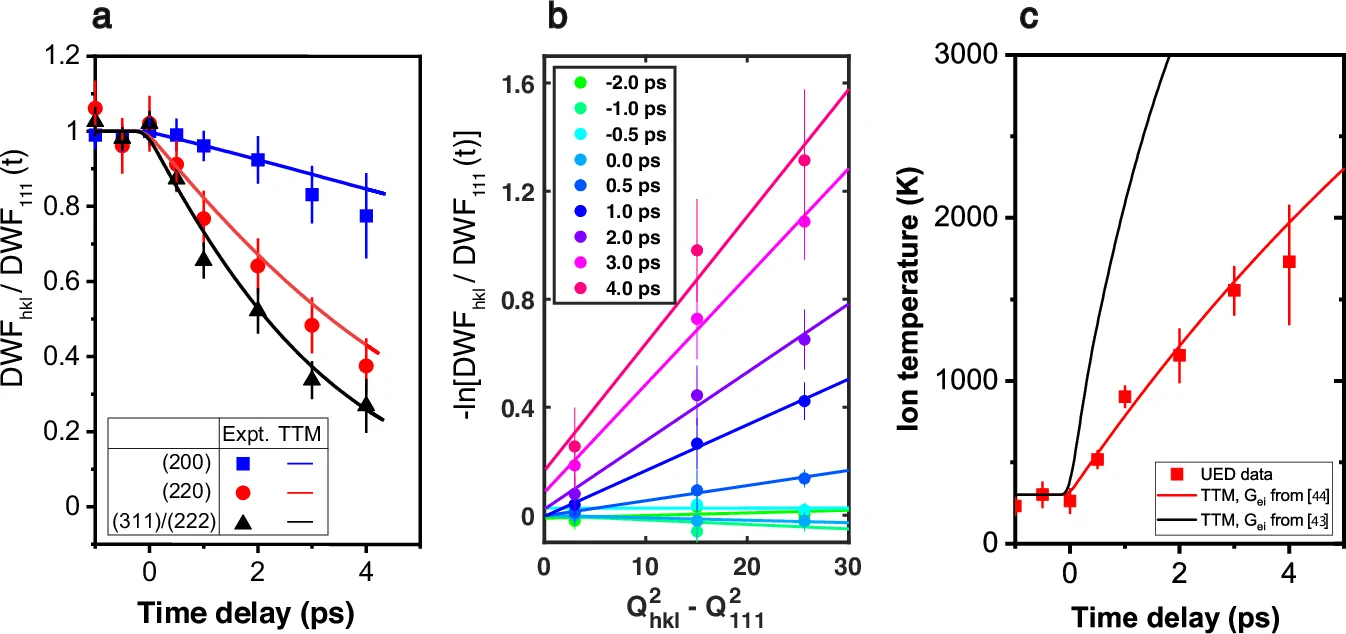
Determining the lattice heating. **a** Experimental data of the decay of normalized diffraction peaks, normalized to the (111) peak (discrete data points), in comparison with corresponding DWFs that are calculated from TTM simulation results (solid lines). In these TTM simulations, we adopted a temperature-dependent *G*_*ei*_ from Migdal et al.^44^ (see “Methods”). **b** Negative logarithm of the normalized DWF (discrete data points) as a function of ${Q}_{\,{\rm{hkl}}\,}^{2}-{Q}_{111}^{2}$, overlaid with linear fits (solid lines) to extract the mean displacement $\Delta \langle {u}^{2}\rangle (t)$. **c** Evolution of lattice temperature derived from the measured DWFs compared to TTM simulation results based on electron-ion coupling according to Lin et al.^43^ (black line), and Migdal et al.^44^ (red line). Data in all graphs are for *ϵ *= 2.55 MJ kg^−1^ with error bars representing one standard deviation.

**Fig. 4:**
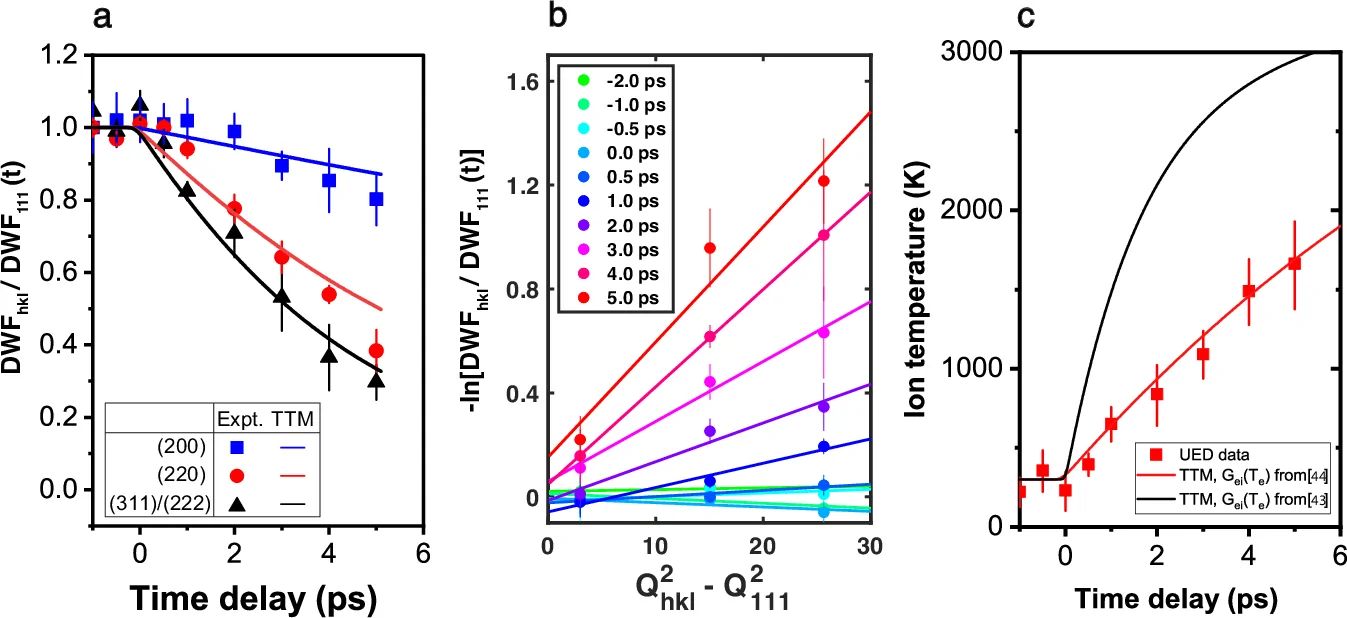
UED measurement of lattice heating in fs laser-heated copper. **a**–**c** present the same measurements and analysis as in Fig. 3, but for a lower absorbed energy density (1.26 MJ kg^−1^). The panels show the evolution of the diffraction peaks, the Debye–Waller analysis, and the lattice temperature extracted from the UED measurements.

We present the DWF results for an energy density of *ϵ *= 2.55 MJ kg^−1^ in Fig. 3a, while the results for *ϵ *= 1.26 MJ kg^−1^ are shown in Fig. 4a. When melting starts, the phase change lowers the fraction of solid phase in the heated film and accordingly results in an additional decay of the solid diffraction peak intensity. This effect is manifested by the emergence of the positive intercept with the linear fit to the logarithm of the DWF at time delays larger than 2 ps as shown in Fig. 3b, which is not expected if it were a sole DWF effect^39^. The time when the deviation starts is also consistent with the onset of melting determined from the width of the (111) peak. At the lower energy density (*ϵ *= 1.26 MJ kg^−1^), the positive intercept appears at a later time due to the delay in the onset of the melting process, c.f. Fig. 4b.

We determine the ion temperature evolution using the measured DWFs and the mean square displacement of copper that accounts for the anharmonic contribution^40,41^ (see “Methods”). For the strong excitation shown in Fig. 3c, the ion temperature increases rapidly from the room temperature to ∼ 1750 K at *t* = 4 ps. This corresponds to an average heating rate of ∼ 3.6 × 10^14^ K s^−1^, implying that the system enters into a regime where homogeneous melting dominates the phase transition^13,42^, consistent with our interpretation for the width of the rising liquid peak.

Importantly, the measured ion temperature evolution allows to test models for the electron-lattice coupling (*G*_*ei*_) in copper. We apply the two-temperature model (TTM) to describe the ion temperature evolution in fs laser-heated copper (see “Methods”), and compare the results with the experimental data. We test two different expressions for *G*_*ei*_ and show the comparison in Fig. 3c. The coupling suggested by Lin et al.^43^ results in lattice temperatures clearly exceeding the measured data, while the model by Migdal et al.^44^ predicts a more gentle increase, which aligns well with the experimental results. The same observation is also found for the weaker excitation (c.f. Fig. 4c). When calculating DWFs with the ion temperature from TTM simulations using *G*_*ei*_ from Migdal et al., we find good agreement with the experimental data for all diffraction peaks and both excitation strengths (see Figs. 3a, 4a).

### TTM-MD simulations

We conduct TTM-MD simulations of ultrafast melting in copper using the LAMMPS code^45^ (see “Methods”) to understand the transient atomic structures during the melting transition. We select an embedded atom method (EAM) potential developed by Sheng et al.^46^ to describe the mutual interaction between copper atoms. This potential accurately reproduces the lattice constant and other key bulk parameters of copper, as summarized in Supplementary Table S1 of Supplementary Information. The initial photo-excitation is modeled as a sudden increase in the electronic temperature. For the energy transfer between the lattice and electrons, we select the model by Migdal et al. that matches the measured lattice temperatures (see Figs. 3c, 4c).

**Fig. 5:**
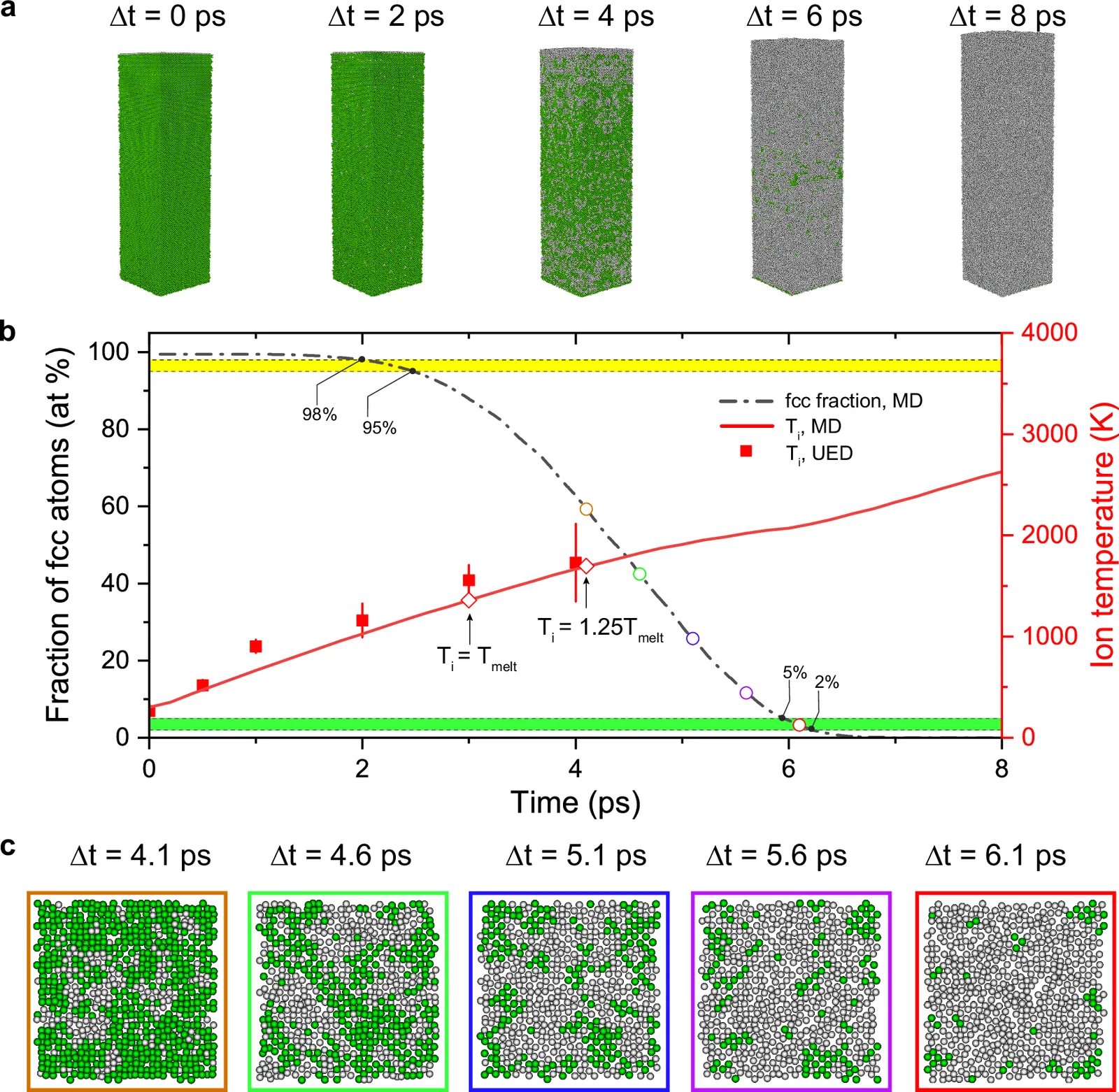
Results of TTM-MD simulation. **a** Snapshots of atomic configurations for different times distributed over the melting transition. The top side of the slab is free to move while the bottom side is fixed to simulate the substrate. The atoms in the simulations are colored according to the surrounding atomic structure: green are atoms in an fcc lattice, gray are disordered (liquid) atoms. **b** Evolution of the fraction of fcc atoms (dash-dot line) and the average ion temperature (red solid line). The yellow band highlights fcc fractions ranging from 98% to 95% (onset of melting); the green band fractions from 5% to 2% (melt completion). The red squares with error bars (1 SD) label lattice temperatures from the electron diffraction measurements for comparison. The energy density considered here is 2.55 MJ kg^−1^. **c** Snapshots of atomic configurations after reaching the predicted superheating limit of 1.25 *T*_melt_ for selected areas at the center of the slab. The time points for the snapshots are marked with color-coded circles on the fcc fraction curve in (**b**).

**Fig. 6:**
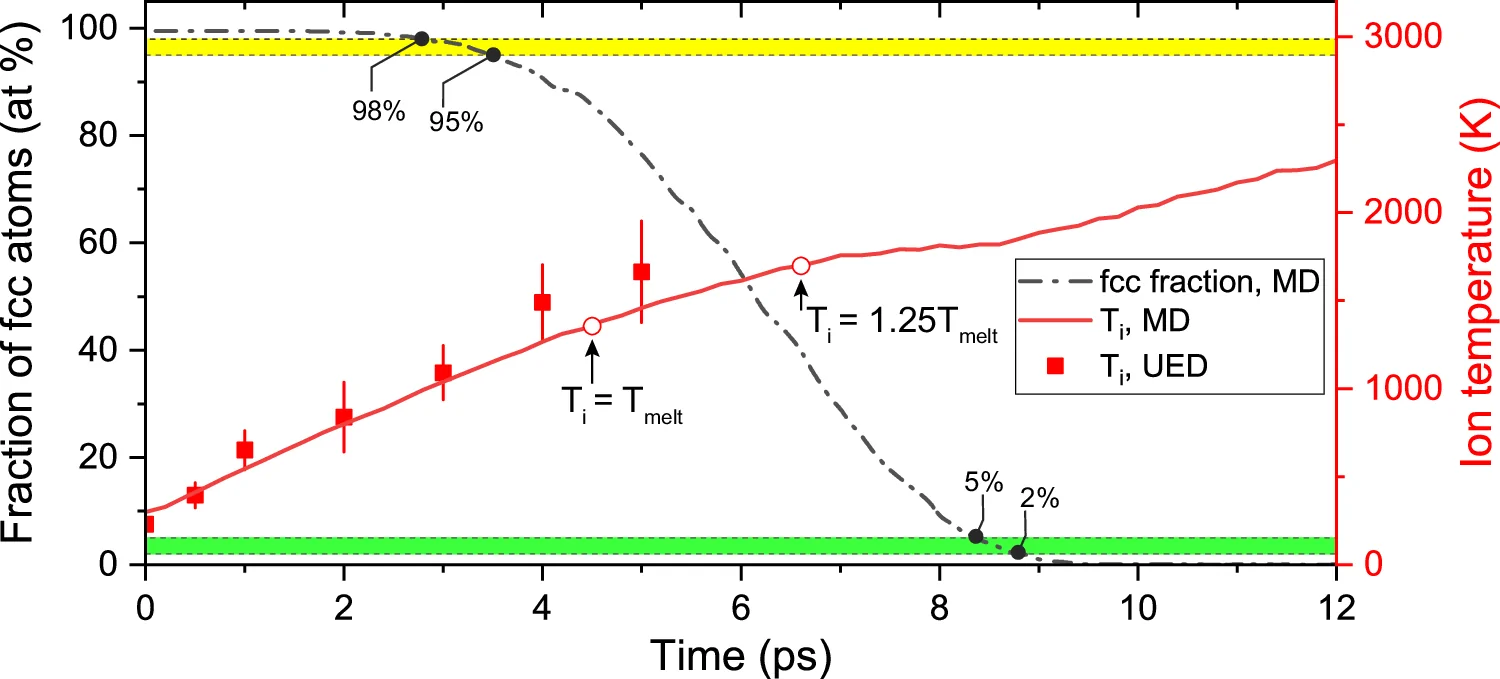
TTM-MD simulation results of ultrafast melting in fs laser-heated copper. The results are presented in the same way as Fig. 5b, but for *ϵ *= 1.26 MJ kg^−1^.

Figure 5 presents the simulation results for the time evolution of the average ion temperature, *T*_*i*_, and the fraction of fcc atoms, *ψ*, for *ϵ *= 2.55 MJ kg^−1^. In addition, snapshots of atomic configurations at different times sampling the solid-to-liquid phase transition are shown. Due to our choice of the electron-lattice coupling, the MD simulation follows the measured lattice temperature for the first 4 ps. Similarly, the simulation results for the case of *ϵ *= 1.26 MJ kg^−1^ are shown in Fig. 6.

The atomic configurations indicate that the film remains nearly crystalline up to 2 ps after excitation, at which time *T*_*i*_ ≈ 1025 K. Shortly afterwards, but before the nominal melting temperature is reached, we observe the onset of melting, marked by a weak decrease in *ψ*. The snapshots show that this decrease in order stems mainly from the free surface at the top of the sample. The transition to atoms not in a fcc lattice strongly accelerates once the melting temperature is reached at roughly 3 ps. From this time onward, we observe that the entire volume contributes to the phase transition in agreement with the prediction of the homogeneous melting regime. At 4 ps, an ion temperature of *T*_*i*_ ≈ 1667 K is reached, and roughly half the sample has transitioned to the liquid phase. Although we have slightly more disordered atoms near the free surface, this part does not contribute substantially to the overall melting process. In particular, we do not observe a melting wave from the surface but a transition to the disordered phase homogeneously distributed throughout the sample once the melting temperature is reached. This process is almost finished around 6 ps.

Due to the fast heating rate, the ion temperature rises steadily far beyond the melting temperatures and also exceeds a proposed stability limit of the lattice^47^, that is ∼ 1.25 *T*_melt_, 4.1 ps after the excitation. At this limit of superheating, a rapid collapse of the crystalline structure is predicted. Interestingly, we do not observe this behavior. In our simulations, homogeneous melting starts earlier and progresses smoothly without any acceleration once this proposed superheating limit is exceeded. Indeed, we only observe changes in slope of the transition rates at 2 ps when melting starts in the entire volume and after 6 ps when almost all atoms have been converted. To further investigate this behavior, we examine local atomic configurations near the center of the film after the superheating limit (see Fig. 5c). We find that small pockets of liquid appear spontaneously from fluctuations at random positions, which then grow further and drive the melting process. These findings point to a smooth transition starting in the bulk near the nominal melting temperature and progressing on a time scale which is defined by the internal nucleation and growth dynamics.

Our results are in stark contrast to the behavior reported in Ref. ^16^. In that study, the gold samples experienced lattice heating rates exceeding 10^15^ K s^−1^ and were reported to retain their crystalline structure at ion temperatures far above the melting point. In contrast, our measurements are conducted at an order-of-magnitude lower heating rate, which is more relevant to ultrafast laser-materials processing^2,7^, and the results show that melting starts near the nominal melting temperature and smoothly evolves until completion.

## Discussion

To provide further insight into the melting dynamics, we examine the energy density dependence of characteristic times associated with ultrafast melting, i.e., the times for melt onset and melt completion as obtained from both electron diffraction measurements and TTM-MD simulations. Experimentally, we define melt onset as the moment when the (111) peak width starts to broaden, and melt completion as the point when the (220) peak disappears (c.f. data in Fig. 2). In the simulations, the gradual nature of the phase transition requires a statistical definition of these times. Adapting the approach by Arefev et al.^47^, we define melt onset as the time when 98–95% of the atoms are in the fcc lattice, and melt completion as the time when this fraction falls in the range of 5-2%.

**Fig. 7:**
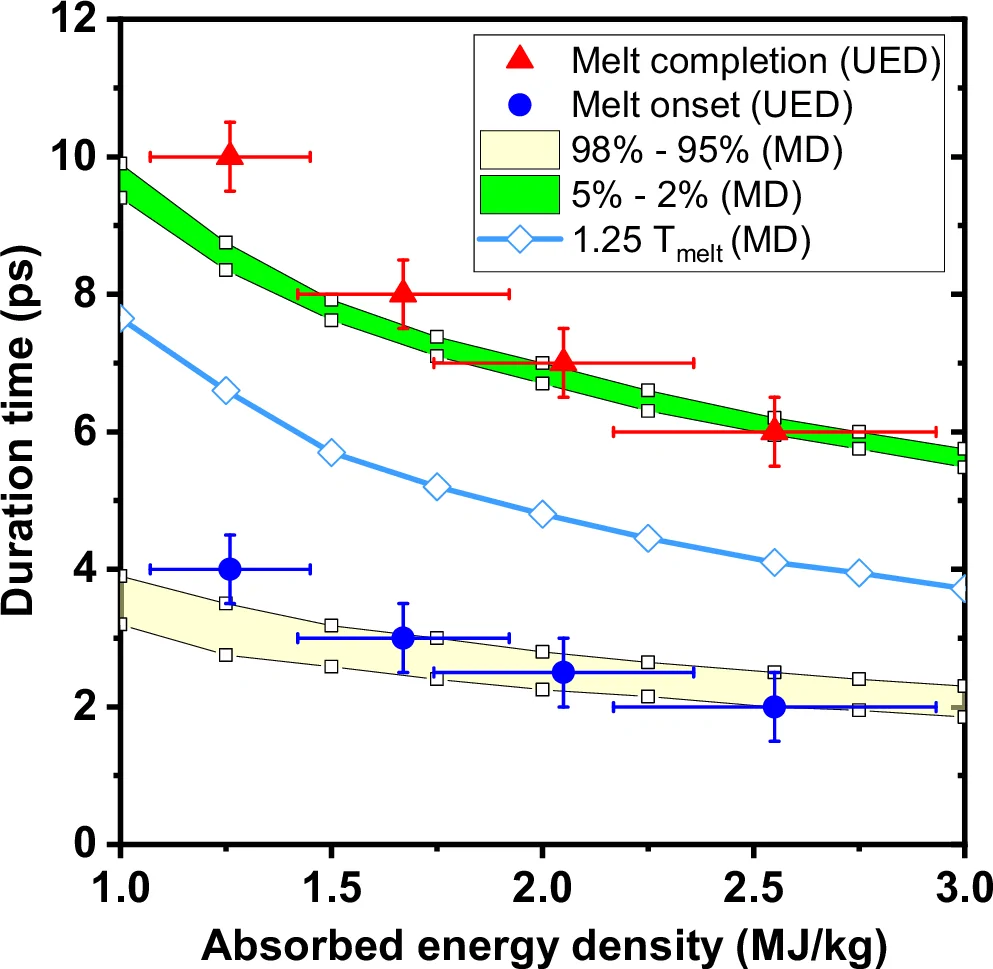
Energy density dependence of melting times for copper. The measured times for melt completion and onset are represented by red triangles and blue circles, respectively. Error bars represent one standard deviation of statistical uncertainties. The data are compared to results from TTM-MD simulations, with open squares indicating the energy densities that have been modeled. The green shaded area corresponds to times when the fraction of fcc atoms is between 5% and 2% (melt completion), the yellow shaded area for times with fcc fractions from 98% to 95% (onset of melting). The light blue line with open diamonds represents the times when the average ion temperature reaches 1.25 *T*_melt_.

Figure 7 demonstrates that the measurements are well reproduced by our TTM-MD simulations for both characteristic times. We also observe that all time scales exhibit a gentle decrease when the target absorbs more energy. This trend is consistent with the results for ultrafast melting of gold within the homogeneous melting regime^13^. However, copper melts slightly faster than gold (10-20 ps for the excitation strength shown) owing to its larger electron-phonon coupling strength.

We also observe that, in all cases, the onset of melt is reached significantly earlier than the superheating limit of 1.25 *T*_melt_ for both simulations and experiments. Indeed, the simulation results show that *T*_*i*_ is just slightly below the nominal melt temperature *T*_melt_ when the material starts to melt (c.f. Fig. 5), consistent with the surface melting effect observed in electron diffraction measurements. Moreover, we don’t observe any special features, like a kink in the transition rates, around 1.25 *T*_melt_ for all cases investigated, although this predicted superheating limit is always crossed between the onset and completion of melting.

We can now construct a physical picture of the energy flow in copper driven by intense short-pulse laser excitations. As an initial condition, the laser pulse deposits the energy to the electrons, which thermalize within the pulse duration^4^. Under our experimental conditions, the initial temperatures of the electrons are approximately two orders of magnitude higher than the ion temperature, forming highly non-equilibrium conditions. The subsequent energy transfer from the electrons to the lattice is determined by the strength of electron-ion coupling, for which strongly deviating predictions have been made^30,31,43,44^. In this study, we track the lattice temperature at early times using the Debye-Waller effect, which dominates the intensity decay of solid diffraction peaks. We find that weak electron-phonon coupling, as suggested by Migdal et al.^44^ agrees well with the experimental temperature evolution, whereas the models predicting a stronger coupling disagree with the data significantly. This finding is further supported by the ion temperatures in the liquid formed after melting, c.f. Supplementary Fig. S1, determined by comparing the measured liquid structure factor with density functional theory (DFT)-MD simulations.

To interpret the melting dynamics, one has to consider the fact that the relatively strong electron-ion coupling in copper leads to rapid lattice heating, allowing the crystal to reach the melting temperature and the superheating limit within a few picoseconds (c.f. Fig. 7). It is the prevailing view that melting within a superheated crystal occurs as a threshold-like process, marked by the rapid and widespread homogeneous nucleation of liquid regions^47^. Contrary to this prediction, our measurements and TTM-MD simulations clearly reveal that melting in the thin copper films begins much earlier, slightly below the nominal melting temperature. This fact cannot be fully explained by the reduced superheating limit resulting from the dynamic relaxation of stresses generated by the ultrafast laser heating^11,47^. Instead, the observed melting onset is likely triggered by melting starting at free surfaces and grain boundaries. However, heterogeneous melting only plays a minor role in the very beginning of the phase transition, and the dominant contribution comes from homogeneous melting that starts at the melting temperature and smoothly transitions over the superheating limit. This interpretation is consistent with prior experiments and MD simulations^13,15,35^ showing that, once the homogeneous melting regime is reached, melting kinetics are largely insensitive to grain size or microstructure. Moreover, our TTM-MD simulations, despite not considering phonon hardening^48,49^, still reproduce both the early-time lattice temperature evolution and the complete melt time. This observation suggests that phonon hardening only plays a minor role under the present experimental conditions.

Previous work by Jourdain et al.^28^ reported characteristic times less than 1 ps for the loss of lattice periodicity in fs laser-heated copper. These results were obtained at comparable energy densities using the technique of X-ray absorption near-edge spectroscopy (XANES). Under the driven conditions, homogeneous melting is the primary mechanism, and hence the difference in sample thickness between the XANES measurement (80 nm) and the current UED experiment (40 nm) cannot account for the discrepancy. Instead, this apparent contradiction reflects the different observables of the two techniques: the transition of electronic structure probed by XANES is more pronounced during melting than that of ionic structure probed by diffraction^28^. These results underscore the importance of directly observing atomic structure and temperature when investigating the dynamics of melting.

Our electron diffraction measurements provided an atomic-level view of ultrafast melting in copper driven by intense femtosecond laser excitations. The pump-probe scheme delivered highly detailed structural information with subpicosecond time resolution, capturing lattice heating and loss of long-range structure as the material transitioned from the cold solid to the liquid state. Through the DWF, we determined the ion temperatures at early times when the target is mostly solid and, thus, could test models for the electron-phonon coupling. We found good agreement with a relatively slow energy exchange rate in copper. Real-time observation of the structural evolution revealed an onset of melting slightly below the nominal melting point, followed by a much faster transition once the melting temperature is reached. We attributed the first effect to melting at free surfaces and grain boundaries, and the acceleration to the initiation of melting throughout the volume of the sample. Our TTM-MD simulations of the ultrafast melting dynamics showed very good agreement with the diffraction data, in particular with respect to the energy density-dependence of melt onset and completion.

Our study provides crucial insights for refining theories of ultrafast melting in metals. In particular, the lack of evidence for a collapse of the lattice structure at the proposed superheating limit challenges the prevailing view that such a collapse should be triggered at this temperature. These results for copper can have strong implications for other metals and advancing materials processing techniques that aim to achieve atomic-level precision.

## Methods

### Experimental details

Our ultrafast pump-probe experiment was performed at the MeV-UED instrument of the Linac Coherent Light Source (LCLS) user facility at SLAC National Accelerator Laboratory ^50,51^. The MeV electrons for diffraction were generated by a Linac Coherent Light Source (LCLS)-type photocathode radio-frequency (RF) gun. The RF gun is powered by a pulse-forming-network based modulator and a 50 MW S-band klystron. Time-resolved diffraction experiments were performed at normal incidence in transmission geometry with relativistic electrons with a kinetic energy of 3.2 MeV. The relativistic electrons were focused by two separated solenoids installed after the RF gun onto the target with diameters of ∼ 120 μm (FWHM), bunch charges of ∼ 20 fC and pulse durations of ∼ 350 fs at FWHM. The electron detector was located 3.2 m away from the sample and consisted of a P43 phosphor screen, a lens system and a sensitive Electron-Multiplying CCD (EMCCD) camera. In the middle of the phosphor screen, a 1.6 mm-diameter through-hole prevented the zero-order diffraction signal from saturating the CCD image at high gain during the experiment.

The samples employed in this study were 40 nm-thick polycrystalline copper films coated on 30 nm-thick, free-standing, amorphous Si_3_N_4_ membranes. These samples were mounted on an array of 350 μm × 350 μm windows supported by a 2" × 1" Si wafer. The samples were uniformly excited from the copper side by focused 400 nm, 130 fs (FWHM) laser pulses at ∼ 4° angle of incidence with a flat-top-like intensity profiles of ∼ 400 μm diameters. The flat-top pump profile was achieved with 10:1 imaging of an aperture outside the target chamber by a single lens with a focal length of 25 cm. The root-mean-square intensity variation was approximately 15% of the averaged value within the full probed area. During our melting study, the pump-probe measurements were conducted in a range of incident laser fluences between 105 mJ cm^−2^ to 210 mJ cm^−2^. In this fluence range, the absorption coefficient of 400 nm laser light was measured to be constant at ∼ 43% in an offline reflection and transmission experiment.

The different excitation states of copper samples were reached by changing the absorbed energy densities *ϵ*, which is defined as the amount of absorbed laser energy over the mass of the excited material and is given by: *ϵ* = (*E*_*inc*_ × *A*)/(*S* × *d* × *ρ*), where *E*_*inc*_ is the measured incident laser pulse energy within the probed area *S*,  *A* is the measured absorption coefficient of the incident laser energy for our samples, *d* is the copper layer thickness, and *ρ* is the mass density for copper. We note here that the thermal energy loss to the substrate is neglected as a first-order approximation due to the short delay time window relevant to this melting study.

### Complete melting threshold

The complete melting threshold is defined as the amount of energy that is required to heat the material from room temperature to its equilibrium melting temperature and to melt the entire film at the melting temperature. This complete melting threshold can be expressed in terms of absorbed energy density, *ϵ*_*m*_, given by :

2 $${\epsilon }_{m}=\int_{300}^{{T}_{{{\rm{melt}}}}}{C}_{i}\,dT+\Delta {H}_{m}$$

where *C*_*i*_ is the ion specific heat capacity, and Δ*H*_*m*_ is the enthalpy of fusion. Note that the electronic contribution to *ϵ*_*m*_ is very small (∼ 1%) and hence it is neglected here. Using the following parameters: Δ*H*_*m *_= 208.7 kJ kg^−1^, *T*_melt _= 1358 K^36^, and *C*_*i*_ = 3.5 × 10^6 ^J/m^3^/K, which was obtained by the Dulong-Petit law, we estimated *ϵ*_*m*_ to be approximately 0.62 MJ kg^−1^ for copper.

### Debye-waller factor and two-temperature model

The attenuation of the diffraction peak intensity can be modeled using the DWF and the temperature evolution results of the TTM simulations. In the TTM framework, electrons and ions are treated as two distinct subsystems, each defined by its own temperature. Thermal equilibrium between the two subsystems is governed by the electron-ion coupling strength *G*_*ei*_. Given that uniform heating is expected under our experimental conditions, electron thermal conduction can be neglected and the model equations are given by:

3a $${C}_{e}({T}_{e})\frac{\partial {T}_{e}}{\partial t}=-{G}_{ei}({T}_{e}-{T}_{i})+S(t)$$

3b $${C}_{i}\frac{\partial {T}_{i}}{\partial t}={G}_{ei}({T}_{e}-{T}_{i})$$

where *T*_*e*_ and *T*_*i*_ are the electron and ion temperature, *S*(*t*) is the temporal profile of the absorbed laser pulse, which is a Gaussian function with FWHM of 130 fs. The electron heat capacity, *C*_*e*_(*T*_*e*_), was calculated from the electronic DOS and the distribution functions^32^. The ion heat capacity, *C*_*i*_, was assumed constant at 3.5 × 10^6 ^J/m^3^/K, which was obtained by the Dulong-Petit law.

With the simulated temperature evolution, the DWF at given *t* and *Q* can be calculated by expression (1), which has been described in detail elsewhere^39^.

We used published data on the temperature dependence of mean square displacement (MSD) for copper to convert the experimental data to the transient ion temperature (c.f. Supplementary Fig. S2 for the case of 2.55 MJ kg^−1^). Specifically, we adopted the data reported in Refs. ^40,41^, which accounts for the anharmonic contribution to DWF derived from high-energy X-ray scattering experiments. The corresponding temperature dependence of the MSD from these references is shown in Supplementary Fig. S3a, and the resulting *T*_*i*_ values are presented in Supplementary Fig. S3b.

For comparison, we also calculated the MSD using the following two models and added the results to Supplementary Fig. S3. The first model is known as the harmonic approximation of the DWF and is given by Ref. ^48^:

4 $$\langle {u}^{2}\rangle=\frac{9{h}^{2}}{4{\pi }^{2}M{k}_{B}{\Theta }_{D}}\left[\frac{1}{4}+{\left(\frac{{T}_{i}}{{\Theta }_{D}}\right)}^{2}\int_{0}^{{\Theta }_{D}/{T}_{i}}\frac{x}{exp(x)-1}dx\right]$$

where *h* is the Planck constant, *Θ*_*D*_ is the Debye temperature (*Θ*_*D *_= 310 K for copper), *k*_*B*_ is the Boltzmann constant and *M* is the atomic mass.

The second model is a fourth-order polynomial parametrization of the equilibrium Debye-Waller B factor^52^. The Debye-Waller B factor is given by:

5 $$B({T}_{i})=\frac{8}{3}{\pi }^{2}\langle {u}^{2}\rangle ({T}_{i})={a}_{0}+{a}_{1}{T}_{i}+{a}_{2}{T}_{i}^{2}+{a}_{3}{T}_{i}^{3}+{a}_{4}{T}_{i}^{4}.$$

For copper, *a*_0_ = 0.08444, *a*_1_ = 0.138 × 10^−2^, *a*_2_ = 0.1185 × 10^−5^, *a*_3_ = − 0.1276 × 10^−8^, and *a*_4_ = 0.4956 × 10^−12^.

As indicated in Supplementary Fig. S3b, the polynomial fit of Debye-Waller B factor shows an overall good agreement with the adopted model that accounts for the anharmonic contribution, while the harmonic model predicts slightly higher temperatures at longer time delays.

To verify the DWF model adopted in this work, we provide the results in Supplementary Fig. S4 that show the temporal evolution of lattice temperature obtained at the two absorbed energy density conditions, i.e., 0.12 MJ kg^−1^ and 0.06 MJ kg^−1^, that are below the damage threshold. The results from UED measurements were inferred using the approach as described in the main text. In addition, we conducted a TTM simulation of the lattice temperature evolution at the same energy density and compared with the experimental result. We find an overall good agreement between the experiment and simulation results for the two conditions, therefore verifying the DWF model employed to infer the lattice temperature evolution in the early stage of fs laser-induced heating in copper.

### Estimate of Lindemann ratio for copper melting

We estimate the Lindemann ratio for copper based on the MSD model employed to extract ion temperature from the DWF analysis.

At the nominal melting temperature of copper (*T*_melt_ = 1358 K), the DWF model adopted in this work predicts a relative change of MSD, $\Delta \langle {u}^{2}\rangle$ = 0.0975 Å^2^ (Supplementary Fig. S3a). Adding the room temperature MSD of 0.0187 Å^2^ gives an absolute MSD of 〈*u*^2^〉 = 0.1162 Å^2^. Using the nearest neighbor distance *d* = 2.56 Å for copper and the Lindemann relation 〈*u*^2^〉 = *δ*^2^*d*^2^, we obtain *δ* ≃ 0.13 as the critical ratio of root-mean-square amplitude of thermal vibration to nearest-neighbor distance at melting. For comparison, the other two DWF models shown in Supplementary Fig. S3a yield *δ* ≃ 0.11.

Reported Lindemann ratios for fcc metals span a range depending on how the MSD is evaluated. Gilvarry et al. reported values around 0.11 for fcc crystals^53^, and values as low as 0.08 for copper when the ratio was inferred from elastic properties by extrapolating the bulk modulus under the assumption of a temperature-independent Poisson ratio, which tends to underestimate the ratio as elastic constants evolve near melting.

Because the present estimate is derived directly from experimentally determined MSD and includes anharmonic contributions, the resulting value of ∼ 0.13 is slightly higher than harmonic or elastic-extrapolation estimates but remains within the commonly reported range of 0.10  −  0.15 for crystalline materials^54^.

### TTM-MD simulations

We carried out atomistic simulations using the approach of the Two-Temperature Model coupled to molecular dynamics simulations for the nuclei (TTM-MD) that is available in the LAMMPS (Large-scale Atomic/Molecular Massively Parallel Simulator) code^45^. The TTM-MD approach adopted in our simulations is based on the work by Duffy and Rutherford^55,56^ and has been developed further to account for adaptive electron background voxel interactions and temperature-dependent coupling parameter^32^.

In the TTM-MD framework, TTM simulates the energy exchange between electronic and atomic subsystems, while MD models the behavior of the heavy particles. The electronic system, modeled as a continuum overlapped with the MD ensemble, was represented using the following heat diffusion equation:

6 $${C}_{e}\left({T}_{e}\right)\frac{\partial {T}_{e}}{\partial t}=\nabla \cdot \left({\kappa }_{e}\nabla {T}_{e}\right)-{G}_{ei}\left({T}_{e}-{T}_{i}\right)+S\left(z,t\right),$$

where *κ*_*e*_ is the electronic heat conductivity, and $S\left(z,t\right)$ is a source term that represents the energy deposited by the laser. To align with the experimental conditions, our simulations assume a uniformly heated target with an electron temperature defined by the deposited energy density as the initial state. Consequently, the source term $S\left(z,t\right)$ can be omitted.

The equations of motion solved by the MD part is

7 $${m}_{i}\frac{\partial {{{\bf{v}}}}_{i}}{\partial t}={{{\bf{F}}}}_{i}-\gamma {{{\bf{v}}}}_{i}+{{{\bf{f}}}}_{L}\left({T}_{e}\right)\,.$$

Here, **v**_*i*_ is the velocity of atom *i* with mass *m*_*i*_. **F**_*i*_ is the classical force acting on the atom calculated using a highly optimized EAM potential developed by Sheng et al.^46^, *γ* represents a frictional drag force, **f**_*L*_ is the stochastic force. The friction and stochastic terms allow energy to be added/removed from the ions to represent energy transfer with electrons, hence *γ* is related to the electron-phonon coupling strength via

8 $$\gamma \,=\left(\frac{V}{{N}^{{\prime} }}\right)\frac{m}{3{k}_{B}}\,{G}_{ei},$$

where *V* is the volume of a coarse grain ionic voxel and ${N}^{{\prime} }$ is the number of atoms in the voxel.

The simulated copper slab has a dimension of 40 nm × 10 nm × 10 nm, comprising a total of 1.35 × 10^5^ copper atoms. The simulation box measures 150 nm × 10 nm × 10 nm and employs periodic boundary conditions in each spatial direction. In order to simulate the effects of substrate interaction and to avoid unphysical pressure reflections of stiff harmonic potentials fixed at the lower side of the slab^32^, 10 layers of substrate atoms are kept at a constant temperature of 300 K (*NVT* ensemble) but allowed to interact with the copper slab through their force potential. Below this region, another region of 5 ghost atom layers are set, which are rigidly kept at their initial position and may only exert an influence on the substrate region through force potential interaction^57^. Using this approach, unphysical pressure or (shock) wave reflections are dampened out. The opposite side of the slab is left free to expand into a vacuum.

The system was first equilibrated for 1 ns at 300 K employing the *NVT* ensemble. The equilibrated configuration was then employed as the initial condition for the TTM-MD simulations conducted under the *NVE* ensemble.

Identification of fcc and non-fcc atoms was performed by means of the compute/ptm package, which is based on the polyhedral template matching algorithm (PTM)^58^. We employed an RMSD cutoff parameter of 0.15 and considered output of fcc, hcp, bcc, ico, and sc structures. In the present study, since the fraction of crystalline structures other than fcc was found to be insignificant (maximum of a few tens of atoms compared to the rest of the ensemble), we can safely attribute the non-fcc copper atoms as liquid atoms in our simulations.

## Supplementary information

Supplementary Information

Transparent Peer Review file

## Data Availability

The UED and MD simulation data generated in this study have been deposited in the FigShare database under accession code : https://doi.org/10.6084/m9.figshare.32840810.

## Code availability

The software used in this study is freely available and can be accessed as follows: LAMMPS.
