# Supplementary material for: Probing the atomic dynamics of ultrafast melting with femtosecond electron diffraction
Source: Nat Commun. 2026 Aug 6;17:9490. doi: 10.1038/s41467-026-75970-1 (PMC13542230; doi:10.1038/s41467-026-75970-1)
Supplement: Supplementary file 1 [file 41467_2026_75970_MOESM1_ESM.pdf]

Supplementary information to ”Probing the atomic dynamics of ultrafast melting with femtosecond electron diffraction”

M. Z. Mo[1,*], M. B. Maigler[2], T. Held[3], B. K. Ofori-Okai[1], A. Bergermann[1,4], Z. Chen[1], R. K. Li[1], X. Shen[1], K. Sokolowski-Tinten[5], R. Redmer[4], X. J. Wang[6,7], J. Schein[2], D. O. Gericke[8], B. Rethfeld[3], and S. H. Glenzer[1]

[1] *SLAC National Accelerator Laboratory, Menlo Park, California, 94025, USA.*

[2] *Bundeswehr University Munich, 85577 Neubiberg, Germany*

[3] *Department of Physics and Research Center OPTIMAS, RPTU Kaiserslautern-Landau, 67663 Kaiserslautern, Germany*

[4] *Institut für Physik, Universität Rostock, 18051 Rostock, Germany*

[5] *Faculty of Physics and Centre for Nanointegration Duisburg-Essen, University of Duisburg-Essen, Lotharstrasse 1, 47048 Duisburg, Germany*

[6] *Faculty of Physics, University of Duisburg-Essen & Research Center Chemical Sciences and Sustainability, 47048 Duisburg, Germany*

[7] *Department of Physics, TU Dortmund University, 44227 Dortmund, Germany*

[8] *Centre for Fusion, Space and Astrophysics, Department of Physics, University of Warwick, Coventry CV4 7AL, United Kingdom*

[*] Corresponding author. Email: mmo09@slac.stanford.edu.

DFT-MD simulations of the liquid structure factor

We performed DFT-MD simulations of the liquid structure factor (LSF) of Cu using the VASP package[1–3], employing the Born-Oppenheimer approximation to decouple the motions of electrons and nuclei. This allowed the nuclei to follow classical trajectories within an effective potential determined by their mutual Coulomb interactions and the electronic charge distribution, which was computed using the Hellman-Feynman theorem. For this simulations, we used projector augmented wave (PAW) potentials [4,5], as implemented in VASP, and applied the Perdew-Burke-Ernzerhof (PBE) formulation of the generalized gradient approximation (GGA) [6]. The Brillouin zone was sampled at the Baldereschi mean value point (BMVP) [7]. To ensure convergence, we used 256 atoms in our simulations with a time step of 1 fs. The Nose-Hoover chain thermostat [8–10] was employed to regulate the temperature of the ions.

We used the simulated LSF to fit the experimental liquid scattering signal to determine the ion temperature after melt completion. Details on the approach have been described elsewhere [11,12]. Briefly, the simulated LSF, denoted by $S(Q)$, was first converted to electron scattering intensity via the following expression : $I(Q) = S(Q)\{f(Q)\gamma\}^2$, where $f(Q)$ is the electron form factor, γ is the Lorentz factor of MeV electrons given by $\gamma = (1-(\upsilon/c)^2)^{-1/2}$ with υ the velocity of the electron and c the speed of light in vacuum. The converted scattering intensity was convoluted with the instrumental function before fitting to the experimental liquid scattering data.

We used the position of the primary liquid peak to first determine the mass density ρ. The ion temperature T_i was subsequently determined by solving for the minimum of the sum of the

relative squared error $\chi^2 = \sum_Q \{I_{sim}(Q,T_i) - I_{exp}(Q)\}^2 / I_{exp}^2(Q)$ between experimental data $I_{exp}(Q)$ and simulation $I_{sim}(Q,T_i)$. In computing the χ^2, we only selected the experimental scattering data located between 2 $\AA^{-1}$ and 8 $\AA^{-1}$ to avoid the interference from the residual of the zero-order diffraction signal at low Q as well as the low signal-to-noise data at very high Q.

In Supplementary Fig. S1, we present the experimental result of the liquid scattering signal along with the best-fit DFT-MD simulation result, for the two time delays of 9 ps (Suppl. Fig. S1a) and 13 ps (Suppl. Fig. S1b) at the absorbed energy density ε = 2.55 MJ/kg. For the liquid scattering data at 9 ps, the mass density and ion temperature are determined to be ρ = 8 g/cm^3 and T_i = 2400 K, as comparing to ρ = 7.75 g/cm^3 and T_i = 3000 K for the time delay of 13 ps. The determined ion temperatures at these two time delays are slightly lower than the TTM-MD predictions, c.f. Suppl. Fig. S1c, which employs the G_{ei} model [13] validated against the early-time ion temperatures determined from DWF. We attribute the discrepancy to the uncertainty ($\sim$ 15%) in the absorbed energy density which was used as an input parameter for the TTM-MD simulation.

Debye-Waller Factor and T_i conversion

A Debye-Waller Factor (DWF) analysis was utilized in this study to determine the ion temperature at early times ($t \leq 4$ ps) of fs laser-induced heating process. To represent the normalized intensity of a Laue diffraction peak measured in UED experiments, the original DWF is modified as [14]:

$$\mathrm{DWF}_{\mathrm{hkl}}(t) = \frac{I_{\mathrm{hkl}}(t)}{I^0_{\mathrm{hkl}}} = \exp\left[-\frac{1}{3}\Delta\langle u^2\rangle(t)\cdot Q^2_{\mathrm{hkl}}\right], \tag{1}$$

where I^0_{hkl} represents the peak intensity before laser arrival, and $\Delta\langle u^2\rangle(t)$ denotes the time-dependent change of the mean squared displacement (MSD) relative to the MSD at ambient conditions. To minimize the effect from pulse-to-pulse fluctuations, we normalize the $\mathrm{DWF}_{\mathrm{hkl}}(t)$ with respect to that of the lowest order peak (111), which yields:

$$\frac{\mathrm{DWF}_{\mathrm{hkl}}}{\mathrm{DWF}_{111}}(t) = \exp\left\{-\frac{1}{3}\Delta\langle u^2\rangle(t)(Q^2_{\mathrm{hkl}} - Q^2_{111})\right\}. \tag{2}$$

Taking the natural logarithm for both sides of Eqn. 2 and move the negative sign to the left hand side yields:

$$-\ln\left\{\frac{\mathrm{DWF}_{\mathrm{hkl}}}{\mathrm{DWF}_{111}}(t)\right\} = \frac{1}{3}\Delta\langle u^2\rangle(t)(Q^2_{\mathrm{hkl}} - Q^2_{111}). \tag{3}$$

The above equation implies that, $\Delta\langle u^2\rangle$ at any time t can be extracted by taking the slope of the linear fit to the logarithm of the inverted DWF as a function of $Q^2_{\mathrm{hkl}} - Q^2_{111}$. We applied this analysis approach to extract $\Delta\langle u^2\rangle(t)$ for the UED measurements conducted in this study. As an example, we show the derived $\Delta\langle u^2\rangle(t)$ for the case of 2.55 MJ/kg in Suppl. Fig. S2. The corresponding linear fits of the normalized DWF's are shown in Fig. 3b of the main text. We then used the published data on the temperature dependence of MSD for copper to convert the experimental data to the transient ion temperature, c.f. Suppl. Fig. S3, the details of which are provided in the Methods section of the main text.

In Suppl. Fig. S4, we show the results on the extracted lattice temperature for laser fluence conditions that are below the damage threshold and compare the results with TTM simulations. We find an overall good agreement between experiment and simulation, therefore verifying the DWF

model employed to infer the lattice temperature evolution in this work. More details are provided in the Methods section of the main text.

Estimation of hydrodynamic expansion time scale

Fast laser excitation leads to a similarly fast rise in pressure. Relaxation of this pressure triggers a rarefaction wave starting at the free surface of the copper film (its thickness is denoted by d) and propagates inward at a speed $c_s = 5$ km/s [15]. With the onset of melting and the partial transition to the liquid state the effective sound velocity is reduced. Therefore, we estimated the time scale of hydrodynamic expansion as $\tau_{\text{expansion}} > d/c_s = 8\,\text{ps}$, which is at least twice the time duration for the early-time DWF analysis.

Electron-phonon coupling factor G_{ei}

The electron-phonon coupling factor, G_{ei}, dictates the energy transfer rate between electrons and phonons in a system under thermal nonequilibrium conditions. For copper, there have been extensive studies on understanding its G_{ei}, both experimentally and theoretically. A comprehensive list of Cu G_{ei} reported in the literature can be found in the study by Medvedev *et al.* [16]. In our work, c.f. Figure 3c and Figure 4c of the main text, we have compared the UED results of the ion temperature evolution in fs-laser heated Cu with TTM simulations based on G_{ei} models obtained by Lin *et al.* [17] and Migdal *et al.* [13], respectively. These two models represent the upper and lower prediction limits at high temperatures, and their T_e-dependence results are shown in Supplementary

Fig. S5.

Sheng EAM Potential

For the TTM-MD simulations conducted in this work, we selected a highly optimized embedded atom method (EAM) potential developed by Sheng *et al.* [18] to describe the mutual interaction between copper atoms. The Sheng EAM potential was developed by fitting the potential-energy surface (PES) derived from first-principle calculations. In addition, the as-derived PESs were shifted and scaled to match experimental reference data. This potential accurately reproduces the lattice constant and other key bulk thermal parameters of copper, as shown in Table S1.

Table S1: Predicted values of the material properties of copper by Sheng EAM potential compared with experimental data. All the values shown here are taken from Ref. 18.

**Property**	**Unit**	**Experiment**	**Sheng EAM**
Lattice constant a	Å	3.615	3.615
Elastic constant c_{11}	GPa	176	175
Bulk modulus k	GPa	140	141
Young's modulus E	GPa	124	125
Melting point T_m	K	1358	1320
Melting enthalpy ΔH_m	kJ/mol	13.3	11.6
Linear thermal-expansion coeff. ε	$10^{-6}\,\mathrm{K}^{-1}$	16.8	16.8

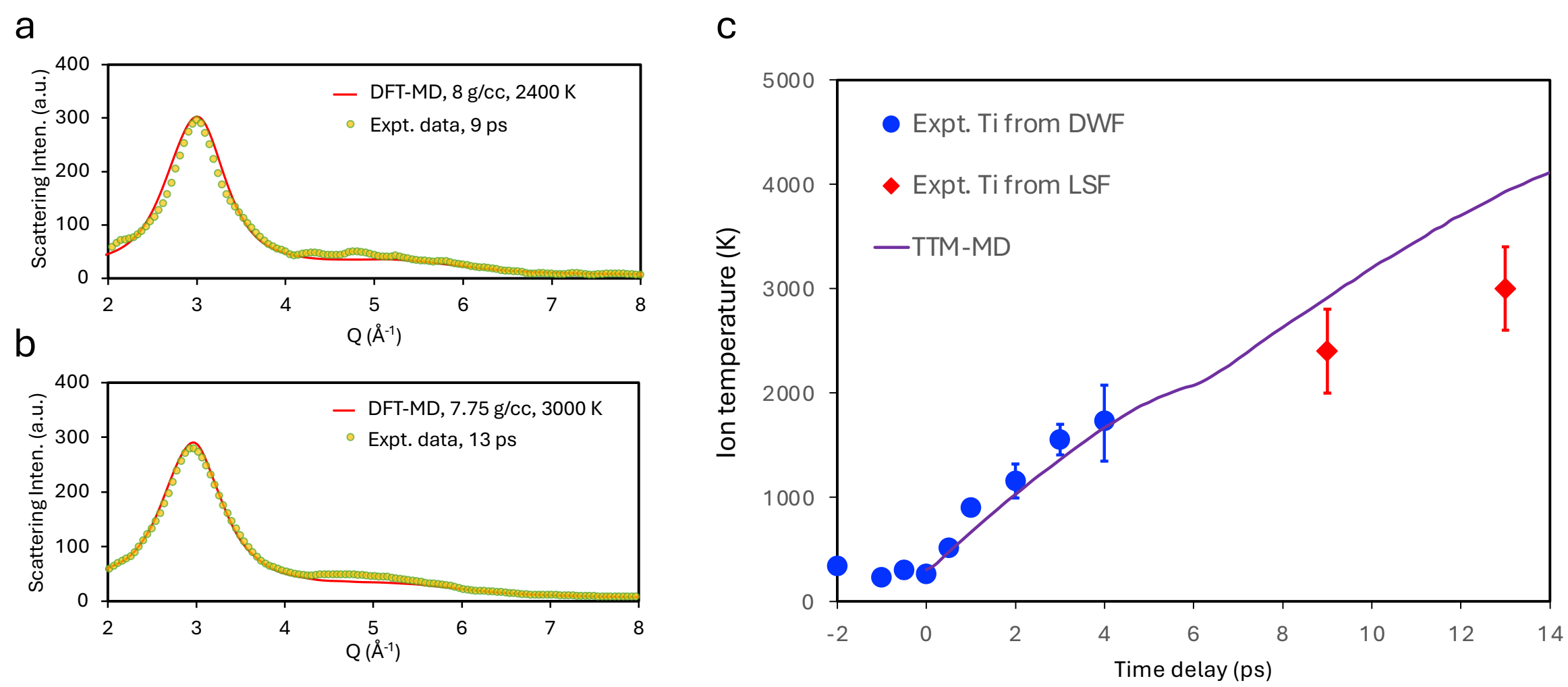

Supplementary Figure S1: Liquid structure factor (LSF) and ion temperature of the molten copper. (a) Liquid scattering signal measured at time delay of 9 ps after the laser excitation (green dotted line). The best fit DFT-MD calculation (mass density $\rho = 8\,\mathrm{g/cm^3}$, $T_i = 2400$ K) is shown as red solid line. (b) The same as in (a) but for a time delay of 13 ps. Corresponding parameters of the best-fit DFT-MD calculation: $\rho = 7.75\,\mathrm{g/cm^3}$, $T_i = 3000$ K. (c) Comparison of the ion temperature evolution between UED measurements and TTM-MD simulation. Blue dots are experimental data extracted from DWF while red diamonds are extracted from LSF. The TTM-MD simulation result shown here is an extended version of the result shown in Figure 5b of the main text. Data in all graphs are for $\varepsilon = 2.55\,\mathrm{MJ/kg}$.

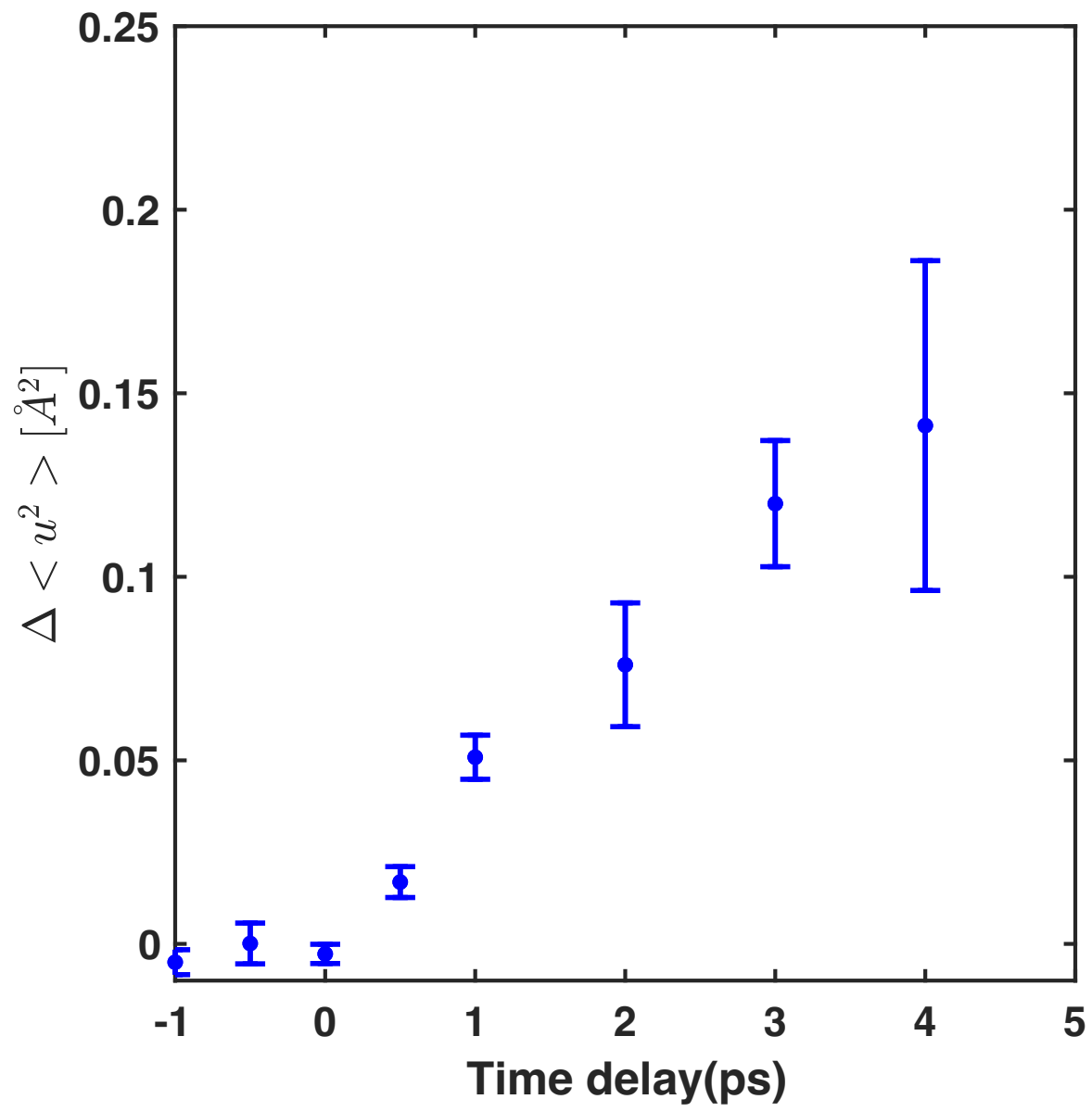

Supplementary Figure S2: Time-dependent change of the MSD in copper after fs laser excitation. These results were extracted from the normalized DWFs for the measurement at $2.55\,\mathrm{MJ\,kg^{-1}}$.

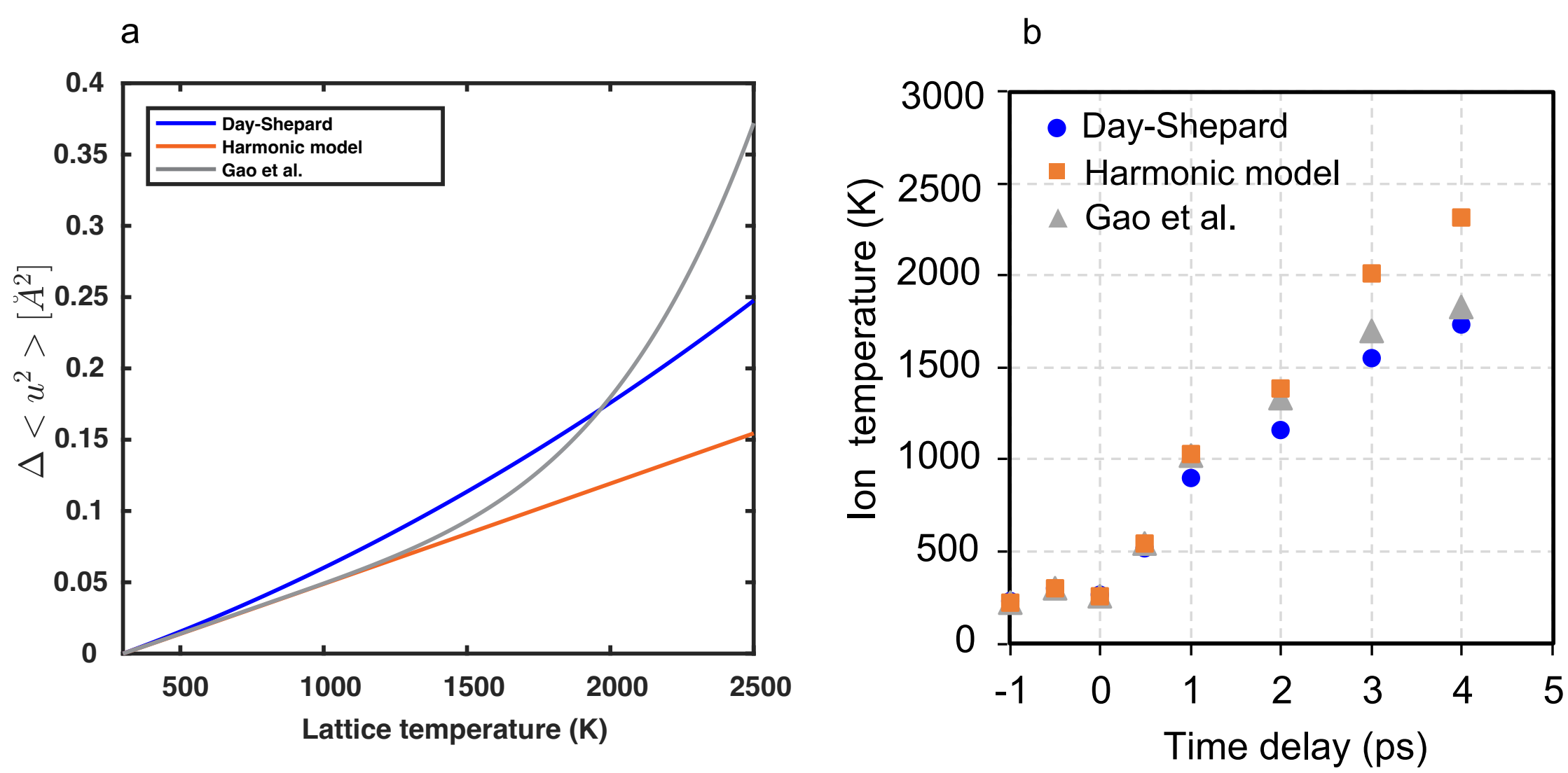

Supplementary Figure S3: Temperature-dependent MSD results of copper estimated with different models. (a) Calculated change of the MSD relative to the RT value as a function of the absolute lattice temperature. The blue line is from a modified DWF model by Day and Shepard *et al.*[19,20] that accounted for anharmonic contribution. The orange line is for the harmonic model, while the gray line is calculated using a forth-order polynomial parameterization of the DWF by Gao *et al.*[21]. (b) Results of the ion temperature derived from the three DWF models as presented in (a). The absorbed energy density is $2.55\,\mathrm{MJ\,kg^{-1}}$.

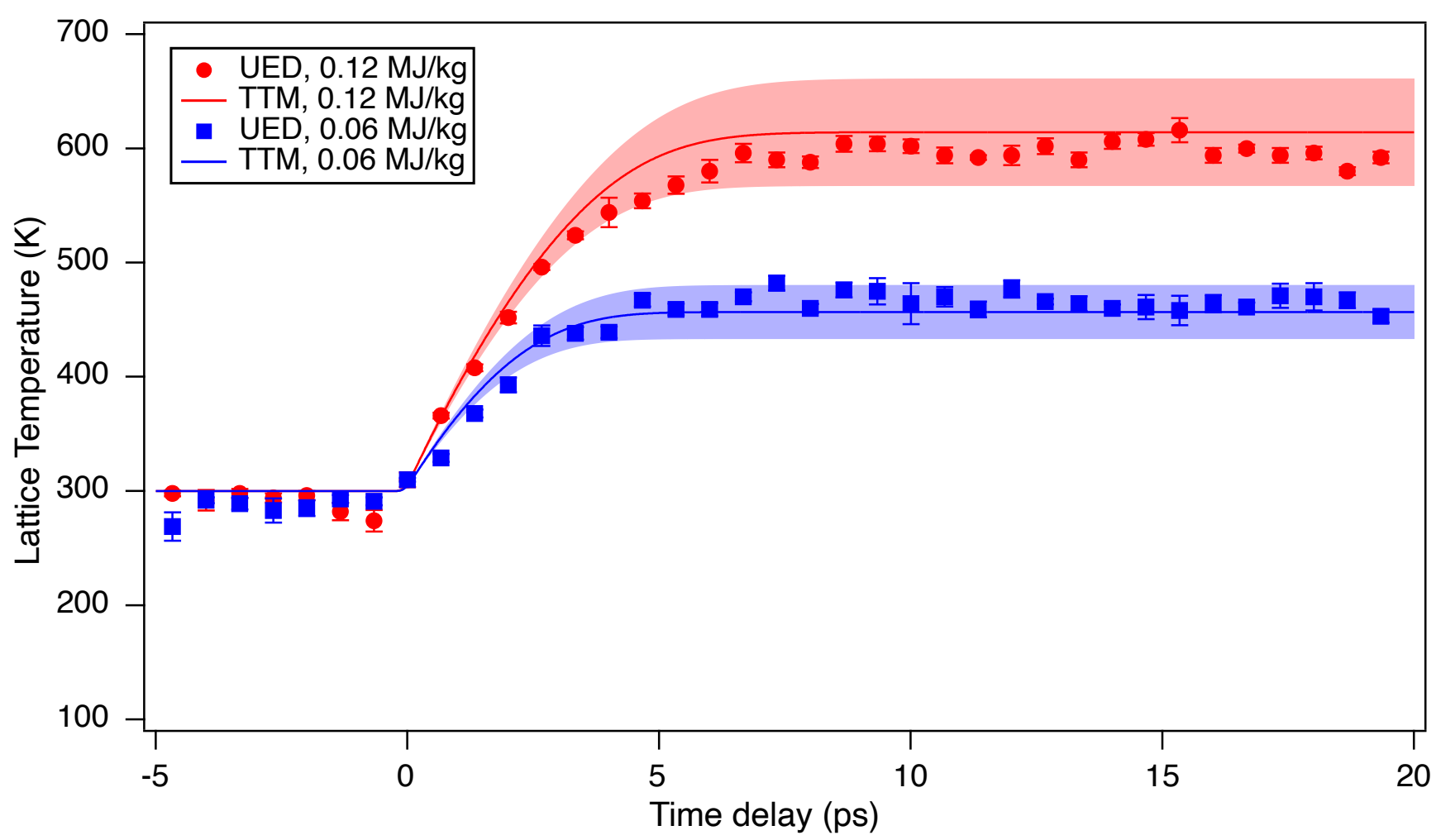

Supplementary Figure S4: Temporal evolution of the lattice temperature in copper excited by femtosecond laser pulses at low fluence conditions that are below the damage threshold. The discrete data points are inferred from the UED measurements using DWF as described in the main text. The red solid circles represent the result for an absorbed energy density of $0.12\,\mathrm{MJ\,kg^{-1}}$ (corresponding incident fluence = $10.1\,\mathrm{mJ\,cm^{-2}}$), and the blue solid squares are for $0.06\,\mathrm{MJ\,kg^{-1}}$ (corresponding incident fluence = $5.2\,\mathrm{mJ\,cm^{-2}}$). The solid lines with shaded areas are TTM simulation results at given energy densities with one standard deviation uncertainty ($\pm15\%$) that corresponds to the intensity non-uniformity of the pump profile.

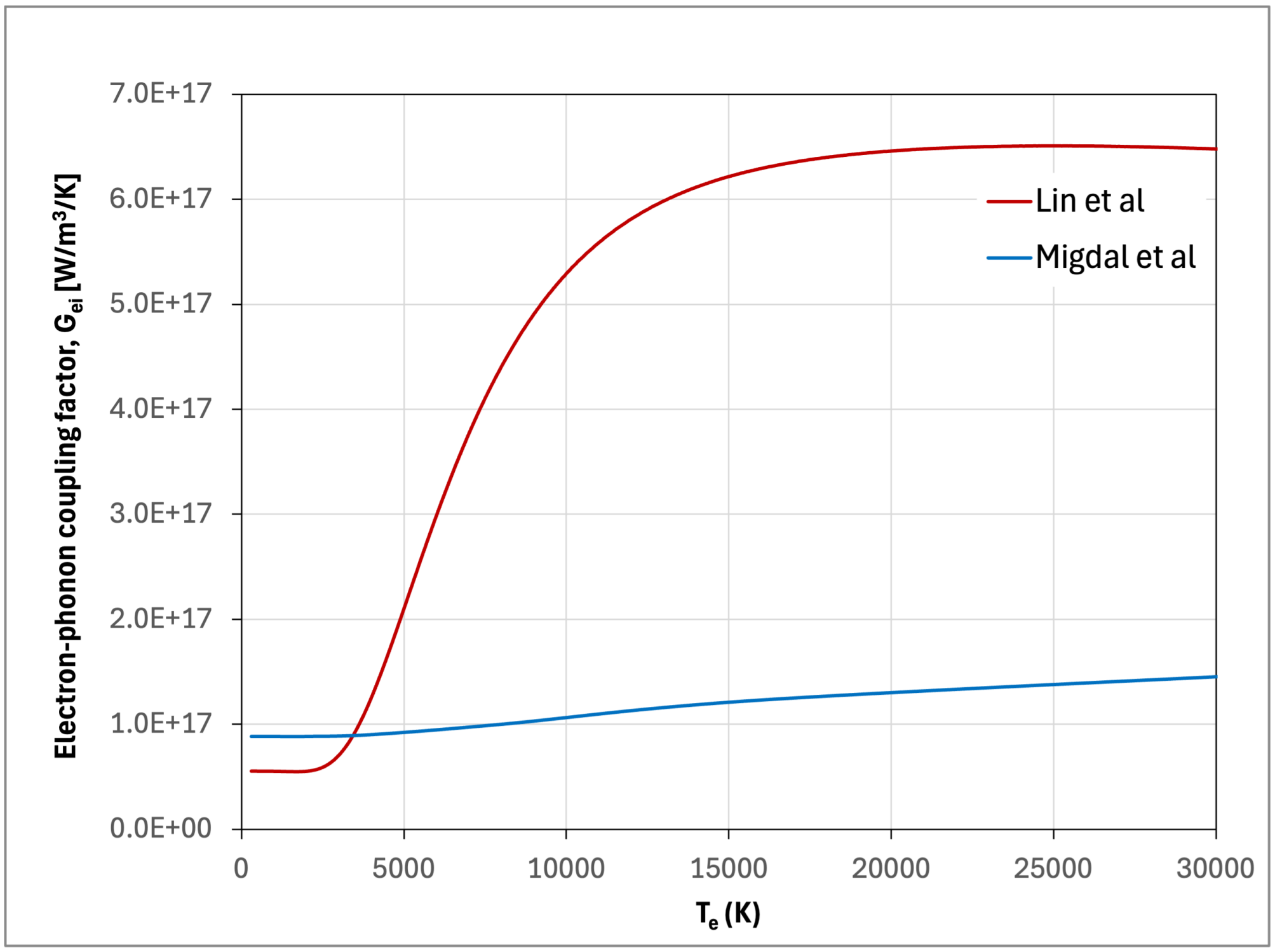

Supplementary Figure S5: T_e-dependence of G_{ei} for Cu. Red line presents the theoretical result by Lin *et al.* [17], while the blue line for the theoretical result by Migdal *et al.* [13].

Supplementary References

1. Kresse, G. & Hafner, J. Ab initio molecular dynamics for liquid metals. *Phys. Rev. B* **47**, 558–561 (1993).

2. Kresse, G. & Furthmüller, J. Efficiency of ab-initio total energy calculations for metals and semiconductors using a plane-wave basis set. *Computational Materials Science* **6**, 15–50 (1996).

3. Hafner, J. Ab-initio simulations of materials using vasp: Density-functional theory and beyond. *J. Comput. Chem.* **29**, 2044–2078 (2008).

4. Blöchl, P. E. Projector augmented-wave method. *Phys. Rev. B* **50**, 17953–17979 (1994).

5. Kresse, G. & Joubert, D. From ultrasoft pseudopotentials to the projector augmented-wave method. *Phys. Rev. B* **59**, 1758–1775 (1999).

6. Perdew, J. P., Burke, K. & Ernzerhof, M. Generalized gradient approximation made simple. *Phys. Rev. Lett.* **77**, 3865–3868 (1996).

7. Baldereschi, A. Mean-value point in the brillouin zone. *Phys. Rev. B* **7**, 5212–5215 (1973).

8. Shuichi, N. Constant temperature molecular dynamics methods. *Prog. Theor. Phys. Suppl.* **103**, 1–46 (1991).

9. Hoover, W. G. Canonical dynamics: Equilibrium phase-space distributions. *Phys. Rev. A* **31**, 1695–1697 (1985).

10. Martyna, G. J., Klein, M. L. & Tuckerman, M. Nosé–hoover chains: The canonical ensemble via continuous dynamics. *J. Chem. Phys.* **97**, 2635–2643 (1992).

11. Mo, M. Z. *et al.* Heterogeneous to homogeneous melting transition visualized with ultrafast electron diffraction. *Science* **360**, 1451 (2018).

12. Mo, M., Chen, Z. & Glenzer, S. Ultrafast visualization of phase transitions in nonequilibrium warm dense matter. *MRS Bulletin* **46**, 694–703 (2021).

13. Migdal, K. *et al.* Heat conductivity of copper in two-temperature state. *Appl. Phys. A* **122**, 408 (2016).

14. Mo, M. Z. *et al.* Determination of the electron-lattice coupling strength of copper with ultrafast MeV electron diffraction. *Rev. Sci. Instrum.* **89**, 10C108 (2018).

15. Jourdain, N., Lecherbourg, L., Recoules, V., Renaudin, P. & Dorchies, F. Electron-ion thermal equilibration dynamics in femtosecond heated warm dense copper. *Phys. Rev. B* **97**, 075148 (2018).

16. Medvedev, N. & Milov, I. Electron-phonon coupling in metals at high electronic temperatures. *Phys. Rev. B* **102**, 064302 (2020).

17. Lin, Z. & Zhigilei, L. V. Time-resolved diffraction profiles and atomic dynamics in short-pulse laser-induced structural transformations: Molecular dynamics study. *Phys. Rev. B* **184113**, 184113 (2006).

18. Sheng, H. W., Kramer, M. J., Cadien, A., Fujita, T. & Chen, M. W. Highly optimized embedded-atom-method potentials for fourteen fcc metals. *Phys. Rev. B* **83**, 134118 (2011).

19. Day, J. T., Mullen, J. G. & Schupp, G. Anharmonic contribution to the debye-waller factor for copper, silver, and lead. *Phys. Rev. B* **93**, 1483 (1994).

20. Shepard, C., Mullen, J. & Schupp, G. Debye-waller factors of copper, silver, and lead. *Phys. Rev. B* **61**, 8622 (2000).

21. Gao, H. X. & Peng, L.-M. Parameterization of the temperature dependence of the debye ± waller factors. *Acta Cryst.* **A55**, 926–932 (1999).
