# Supplementary material for: Probing the atomic dynamics of ultrafast melting with femtosecond electron diffraction
Source: Nat Commun. 2026 Aug 6;17:9490. doi: 10.1038/s41467-026-75970-1 (PMC13542230; doi:10.1038/s41467-026-75970-1)
Supplement: Supplementary file 2 [file 41467_2026_75970_MOESM2_ESM.pdf]

nature portfolio

Peer Review File

Probing the atomic dynamics of ultrafast melting with femtosecond electron diffraction

Corresponding Author: Dr Mianzhen Mo

This file contains all reviewer reports in order by version, followed by all author rebuttals in order by version.

Version 0:

Reviewer comments:

Reviewer #1

(Remarks to the Author)
It is claimed that no indications of rapid lattice collapse were detected and discussed on p12. This is the same message as the earlier Nature paper by some of the same group (White et al [16]). This part of the work simply duplicates the earlier result and is only referenced collectively in the introduction.

The current submission cannot be published in its current form for two major reasons:

1. It requires a full discussion of the important result of ref [16] that the lattice is preserved upon superheating. Since this is one of the main new results presented here, it has to be rationalised fully with the previous work.

2. The case for homogeneous melting needs to be stated much more clearly. As far as this reviewer can see, there is nothing in the new UED measurements that could not be explained by progressive melting initiating from defects where the electron-lattice interaction is favoured. One way to address this would be to make comparative measurements on samples with different grain sizes, but not as performed here.

Once these have been addressed, the authors may wish to correct these smaller points:

p8 "onset of melting τ_onset, the lattice temperature is approximately 1000 K for the stronger excitation (see Fig. 4b), amounting to~75 % of the nominal melting" must be depending on some model since no temperature can be measure.
No estimate for lower fluence - should provide this as check of assumptions.

p9 "well described by exponential growth." there is a clear second component to the trend which is not explained. This may be related to the mixing of crystal Bagg and liquid peaks at different Q positions.

p10 please explain why an "exponential form and the comparable time scales are indicative of homogeneous melting"

p10 ion temperature evolution is delegated to the SI. This essential calculation, and the assumptions underlying it, are required in the main paper.

p11 "highly optimised embedded atom method (EAM) potential" How was the potential optimised? How well does it simulate the lattice constant and key bulk thermal parameters like thermal expansion, melting point of bulk Cu? This information is essential in the manuscript text, even if it is referenced in [46].

p11 "film" is mentioned. Please give simulation sample dimensions of the LAMMPS simulation.

Reviewer #2

(Remarks to the Author)
In this paper, the authors investigate the ultrafast atomic-scale dynamics of laser-inducing melting in Copper using ultrafast electron diffraction, and supported their findings via molecular dynamics calculations. Despite the topic is not a pressing or urgent matter, it is of quite general interest and constantly studied in the literature over the last decades.
The experimental and theoretical data are of high quality, and the work and analysis have been conducted in a thorough manner. However, there are a few issues that the authors should address before publication:
1) Can the authors present long-time scale dynamics to attest to the reversibility of the transformation observed by the authors?
2) the authors have not reported any estimation of the Lindemann limit for Copper, which should be compared with the experimental data
3) It is not clear how the authors can rule out possible strain effects in taking into account the lag time for the FWHM of the diffraction peak (3 ps). Can the authors further comment on this issue?
4) It is not clear the physical argument behind the analysis in Figure 3 and why the peak (111) was chosen. Also, no error bars are included. Can the authors further comment on this issue?

Overall, I believe that the manuscript might potentially be published provided that the authors gives precise answers to my previous comments.

Version 1:

Reviewer comments:

Reviewer #1

(Remarks to the Author)
I am happy with the responses to my questions. I had not appreciated that the fluences used here were beyond to point where heterogeneous melting is seen. The revised version clarifies this.

The work can now be published.

Reviewer #2

(Remarks to the Author)
I have carefully read the response letter by the authors and the revised manuscript. They have clarified all the open issues and questions and therefore I recommend the manuscript for publication.

Open Access This Peer Review File is licensed under a Creative Commons Attribution 4.0 International License, which permits use, sharing, adaptation, distribution and reproduction in any medium or format, as long as you give appropriate credit to the original author(s) and the source, provide a link to the Creative Commons license, and indicate if changes were

made.
In cases where reviewers are anonymous, credit should be given to 'Anonymous Referee' and the source.
The images or other third party material in this Peer Review File are included in the article’s Creative Commons license, unless indicated otherwise in a credit line to the material. If material is not included in the article’s Creative Commons license and your intended use is not permitted by statutory regulation or exceeds the permitted use, you will need to obtain permission directly from the copyright holder.
To view a copy of this license, visit https://creativecommons.org/licenses/by/4.0/

Response to Reviewer 1:

We thank the reviewer for the valuable comments that helped us to improve our manuscript. We have addressed all the concerns and complied with the suggested changes from the reviewer. Our detailed responses are found below.

From reviewer's report:
It is claimed that no indications of rapid lattice collapse were detected and discussed on p12. This is the same message as the earlier Nature paper by some of the same group (White et al [16]). This part of the work simply duplicates the earlier result and is only referenced collectively in the introduction.
The current submission cannot be published in its current form for two major reasons:
1. It requires a full discussion of the important result of ref [16] that the lattice is preserved upon superheating. Since this is one of the main new results presented here, it has to be rationalised fully with the previous work.

Response: We thank the reviewer for the comment and the opportunity to discuss the relevance between the present findings and the work of Ref. [16] (White et al.). However, with all due respect, we have to disagree with the reviewer that the present result duplicates Ref. [16].

The current experiment differs strongly from the one reported in Ref. [16], both in their experimental setups and, more importantly, in the the observed results. In Ref. [16], the gold foils didn't melt at ion temperatures over 14 times the melting point (T_{melt}). Only far beyond this point melting was observed but the resolution of the experiment doesn't allow to estimate if this was due to rapid lattice collapse or slower. In contrast, our measurements show that the melting begins near the nominal melting temperature and then smoothly continues with increasing ion temperature until the entire sample is in the fluid phase. These observations point to a fundamentally different behaviour. Although both experiments didn't see any strong changes at the proposed critical temperature of 1.25 T_{melt}, the outcomes remain qualitatively and quantitatively different: Ref. [16] reports a well-preserved solid at this temperature, whereas our results show partial melting that continues to evolve smoothly with increasing temperature.

We can only speculate why these differences arise. Different material properties (copper vs. gold) are possible. A second possibility is the different heating rates employed in the two experiments. In Ref. [16], the employed laser fluences were much higher resulting in heating rates exceeding $3 \times 10^{15} K s^{-1}$. Under these experimental conditions, the surface- and defect-mediated melting pathways are strongly suppressed. In contrast, the present work operates at an order-of-magnitude lower heating rate that is more relevant to ultrafast laser-materials processing [Refs. 2 and 7] and the results show signs of surface melting in the early time of the heating process.

To address this concern, we have added the following discussion after the last paragraph of page 12 in the revised manuscript to explicitly relate our results to the work of White et al.:

Our results are in stark contrast to the behavior reported in Ref. [16]. In that study, the gold samples experienced lattice heating rates exceeding 10^{15} K s^{-1} and were reported to retain their crystalline structure at ion temperatures far above the melting point. In contrast, our measurements are conducted at an order-of-magnitude lower heating rate, which is more relevant to ultrafast laser-materials pro-

cessing [2, 7], and the results show that melting starts near the nominal melting temperature and smoothly evolves until completion.

> 2. *The case for homogeneous melting needs to be stated much more clearly. As far as this reviewer can see, there is nothing in the new UED measurements that could not be explained by progressive melting initiating from defects where the electron-lattice interaction is favoured. One way to address this would be to make comparative measurements on samples with different grain sizes, but not as performed here.*

Response: We thank the reviewer for raising this point. While comparative measurements on samples with different grain sizes would provide a direct experimental test, several other experimental and computational studies indicate that, once the homogeneous melting regime is reached, melting kinetics become largely insensitive to microstructure.

First, in our previous UED study of laser-induced melting in gold [Ref.13, Science 360, 1451 (2018)], we explicitly compared single-crystal and polycrystalline films with substantially different grain sizes. Under homogeneous melting conditions, we observed similar melting timescales for both sample types, indicating that the nanocrystalline structure has a minor influence on the melting dynamics in this regime.

Second, a theoretical study by Lin et al. [Ref.35, J. Phys. Chem. C 114, 5686 (2010)] reported consistent behavior. Through MD simulations, they examined the influence of nanocrystalline structure on the melting process in gold induced by ultrafast laser excitation. In the high-fluence regime where homogeneous melting dominates, the simulations showed that grain boundaries in nanocrystalline films lead to only a modest reduction in grain size at early times, followed by rapid melting that proceeds on essentially the same timescale as in single-crystalline films.

Lastly, similar conclusions were reached in a combined experimental and simulation study of palladium by Antonowicz et al. [Ref.15, Acta Materialia 276, 120043 (2024)], which reported that surfaces and grain boundaries play only a minor role once homogeneous melting dominates.

Taken together, these results support our interpretation that the dynamics observed in the present UED measurements are consistent with homogeneous melting, even though only polycrystalline samples were studied. Grain-size effects are expected to contribute primarily during the initial laser heating stage, but do not significantly influence the overall melting timescale in the homogeneous melting regime.

To address this concern, we have added the following discussion (highlighted by blue) in the first paragraph of page 15 in the revised manuscript:

”... However, heterogeneous melting only plays a minor role in the very beginning of the phase transition and the dominant contribution comes from homogeneous melting that starts at the melting temperature and smoothly transitions over the superheating limit. This interpretation is consistent with prior experiments and MD simulations [Lin2010, Mo2018,Antonowicz2024] showing that, once the homogeneous melting regime is reached, melting kinetics are largely insensitive to grain size or microstructure. Moreover, our TTM-MD simulations...”

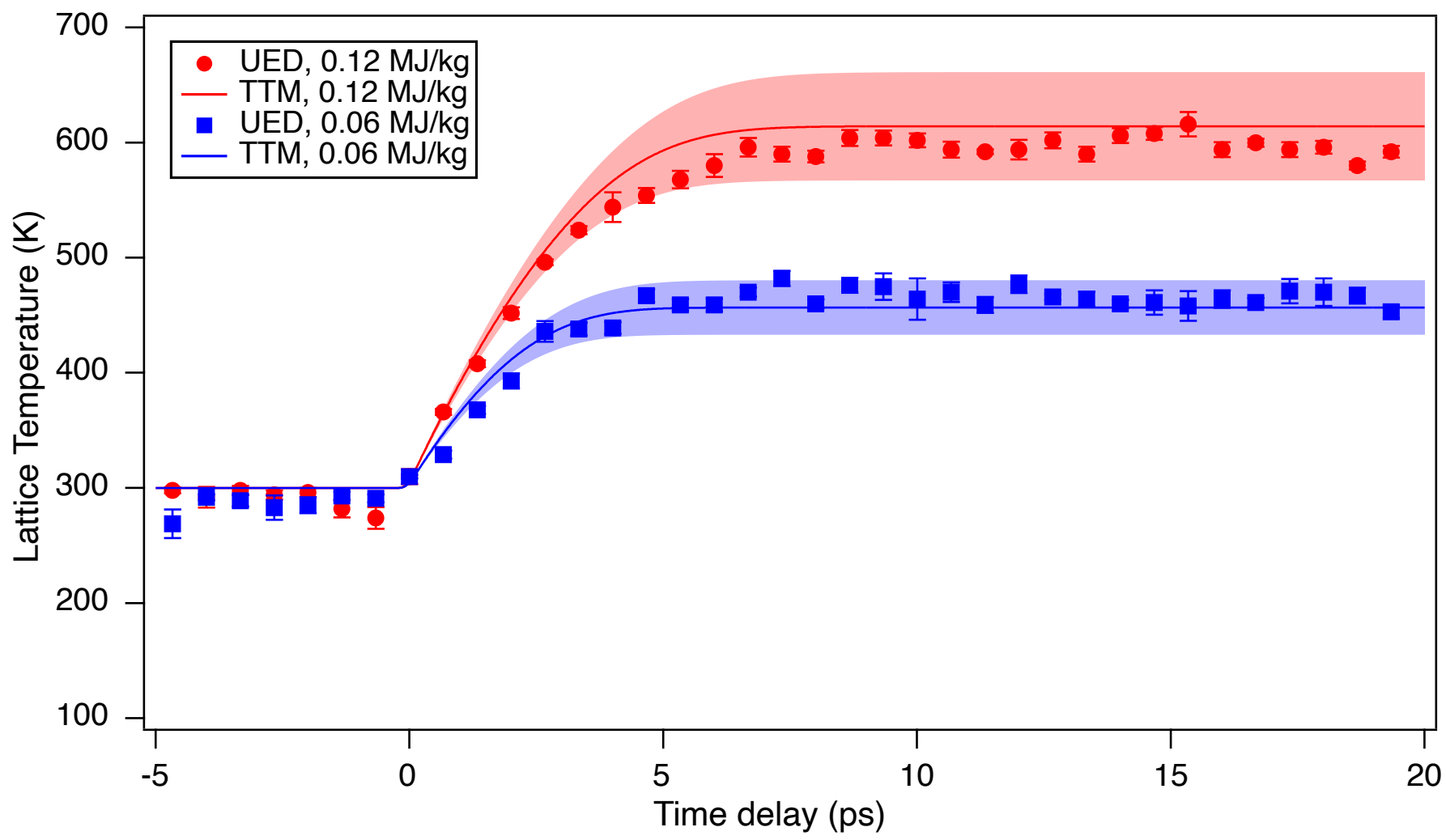

Figure R1: Temporal evolution of the lattice temperature in copper excited by femtosecond laser pulses at low fluence conditions that are below the damage threshold. The discrete data points are inferred from the UED measurements using Debye-Waller factor as described in the main text. The red solid circles represent the result for an absorbed energy density of 0.12 MJ/kg (corresponding incident fluence = 10.1 mJ/cm^2), and the blue solid squares are for 0.06 MJ/kg (corresponding incident fluence = 5.2 mJ/cm^2). The solid lines with shaded areas are TTM simulation results at given energy densities with one standard deviation uncertainty ($\pm 15\%$) that corresponds to the intensity non-uniformity of the pump profile.

Once these have been addressed, the authors may wish to correct these smaller points:
p8 "onset of melting τ_{onset}, the lattice temperature is approximately 1000 K for the stronger excitation (see Fig. 4b), amounting to 75% of the nominal melting" must be depending on some model since no temperature can be measure.
No estimate for lower fluence - should provide this as check of assumptions.

Response: The reviewer is correct that the lattice temperature reported in this work is reliant on a model. This model is known as the Debye-Waller factor used to describe the attenuation of diffraction peak intensity due to thermal heating of the lattice system. Detailed description of the model have been provided in the Methods and Supplementary Information of the original manuscript.

Regarding the latter comment, we have provided lower fluence results in the revised manuscript to verify the model assumption as suggested by the reviewer. For convenience, we provide the results in Figure R1 that show the temporal evolution of lattice temperature obtained at the two absorbed energy density conditions, i.e., 0.12 MJ/kg and 0.06 MJ/kg, that are below the damage threshold. The temperature results from UED measurements (discrete data points) are inferred with the Debye-Waller factor as described in the main text as well as in Ref. 39. In addition, we also conduct TTM simulations of the lattice temperature evolution at the same energy density and compare with the experimental result. As shown, we find an overall good agreement between the experiment and simulation results for the two conditions, therefore verifying the model assumption that we employ to infer the lattice temperature in the early stage of fs laser-induced heating in copper.

Following the suggestion, we added Figure R1 as Extended Data Figure 5 in the revised manuscript, along with the discussion of the figure in Methods, under the subsection of "Debye-Waller Factor and two-temperature model" [Page 21].

> *p9 "well described by exponential growth." there is a clear second component to the trend which is not explained. This may be related to the mixing of crystal Bagg and liquid peaks at different Q positions.*

Response: We agree with the reviewer that the growth of the (111) peak width is related to the mixing of solid Bragg and liquid peaks at different Q positions. This is the exact reason why we select the (111) peak width to track the onset and timescale of liquid formation, as explained in the first paragraph of page 8. However, we do not explicitly extract the second component of the trend, as our goal is to apply a consistent analysis framework across all observables. Specifically, we employ the same fitting function used for the solid Bragg intensity and the diffuse scattering, as given by $1 + A(1 - \exp(-t/\tau))$, and focus on comparing the characteristic time constants extracted from different signals.

To clarify this point, we have made the following changes (highlighted by blue) in the revised manuscript (page 8):

"After melting has started, the width of the (111) peak can be approximated by exponential growth. A second trend may be present during the melting process and could arise from the evolving mixing of solid diffraction peak and liquid scattering at different Q positions. However, for consistency with the analysis of the diffraction peak and diffuse scattering signal, we focus on the extracted time constants rather than the detailed evolution. Nonetheless, in contrast to the decay of the (220) peak, both cases here exhibit similar time constants of approximately (7.5 ±1.0) ps, despite differences in the final equilibrated values at late delay times..."

> *p10 please explain why an "exponential form and the comparable time scales are indicative of homogeneous melting"*

Response: We thank the reviewer for raising this point and would like to clarify the connection between the observed exponential trend and the homogeneous melting mechanism.

For context, homogeneous melting in a superheated crystal is governed by the liquid nucleation rate R within the bulk, which can be expressed as [Ref. 47 : Arefev et al., Sci. Adv. 8, eabo2621 (2022)]:

$$R = R_0 \exp(-\frac{\Delta G}{K_B T})$$

where R_0 is a kinetic prefactor and ΔG is the free-energy barrier for liquid nucleation. Once sufficient superheating is reached, this nucleation process leads to the rapid formation of liquid nuclei throughout the crystal volume, resulting in an exponential reduction of the coherent crystalline domain size.

In contrast, heterogeneous melting proceeds via the propagation of a melt front originating from defects, free surfaces, or grain boundaries. In this regime, the characteristic length scale evolves approximately linearly in time, reflecting a roughly constant melt front velocity. Because the diffraction peak width is inversely proportional to the crystalline domain size, as described by the Scherrer relation, homogeneous bulk nucleation naturally manifests as an exponential increase in peak width, whereas melt-front–driven melting would instead be expected to produce a linear temporal trend. While this framework explains why the dominant behavior follows an exponential form, we note that an additional component may be present in the data. This small deviation could come from the growth of liquid nuclei as well as mixing with the liquid diffraction peak. Nonetheless, this does not alter our interpretation that the observed trend is indicative of homogeneous melting.

Table 1: Predicted values of the material properties of Cu by Sheng EAM potential compared with experimental data. All the values shown here are taken from Ref. 46 [Sheng et al., PRB 83, 134118 (2011)].

**Property**	**Unit**	**Experiment**	**Sheng EAM**
Lattice constant a	Å	3.615	3.615
Elastic constant c_{11}	GPa	176	175
Bulk modulus k	GPa	140	141
Young's modulus E	GPa	124	125
Melting point T_m	K	1358	1320
Melting enthalpy ΔH_m	kJ/mol	13.3	11.6
Linear thermal-expansion coeff. ε	$10^{-6}\,\mathrm{K}^{-1}$	16.8	16.8

p10 ion temperature evolution is delegated to the SI. This essential calculation, and the assumptions underlying it, are required in the main paper.

Response: Following the reviewer's suggestion, we moved the relevant information from SI to the Methods section of the main text in the revised manuscript, i.e., under the subsection of "Debye-Waller Factor and two-temperature model". The original Supplementary Figures 2 and 3 are moved to the main text as Extended Data Figures 3 and 4, respectively.

p11 "highly optimised embedded atom method (EAM) potential" How was the potential optimised? How well does it simulate the lattice constant and key bulk thermal parameters like thermal expansion, melting point of bulk Cu? This information is essential in the manuscript text, even if it is referenced in [46].

Response: The Sheng EAM potential was developed by fitting the potential-energy surface (PES) derived from first-principle calculations [Ref.46]. In addition, the as-derived PESs were shifted and scaled to match experimental reference data. For copper, the values of the material properties predicted by the Sheng EAM potential match well with the experimental data, as shown in Table 1.

Following the reviewer's suggestion, we have made the following changes in the revised manuscript and supplemental information.

Page 11 of the manuscript:

"We select an embedded atom method (EAM) potential developed by Sheng et al. [46] to describe the mutual interaction between copper atoms. This potential accurately reproduces the lattice constant and other key bulk parameters of copper, as summarized by Table S1 of Supplementary Information. The initial photo-excitation is modeled...".

Page 6 of Supplementary information:

We add a section on "Sheng EAM potential" and include Table 1 into the section.

p11 "film" is mentioned. Please give simulation sample dimensions of the LAMMPS simulation.

Response: The sample dimensions [40 nm × 10 nm × 10 nm] of the LAMMPS simulation are provided on page 21 of the original manuscript.

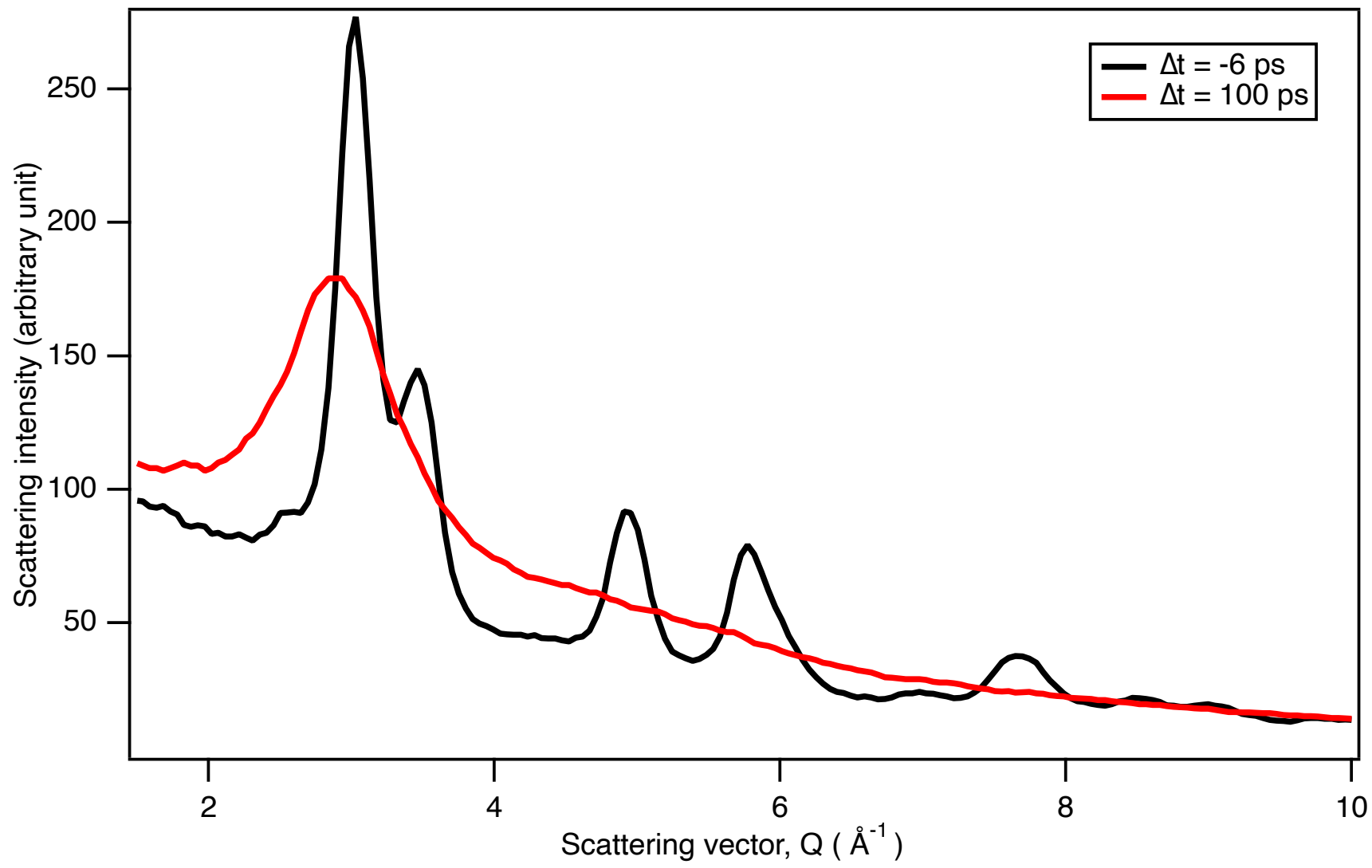

Figure R2: Measured scattering intensity lineout for a delay time $\Delta t = 100$ ps (red solid line) at an absorbed energy density of 2.55 MJ kg^{-1}. For reference, the lineout for $\Delta t = -6$ ps is also shown.

Response to Reviewer 2:

We thank the reviewer for the valuable comments that helped us to improve the quality of our manuscript. We also thank the reviewer for recognizing the significance of our results. We have addressed all the comments from the reviewer and modified our manuscript accordingly. Our detailed responses are found below.

In this paper, the authors investigate the ultrafast atomic-scale dynamics of laser-inducing melting in Copper using ultrafast electron diffraction, and supported their findings via molecular dynamics calculations. Despite the topic is not a pressing or urgent matter, it is of quite general interest and constantly studied in the literature over the last decades.
The experimental and theoretical data are of high quality, and the work and analysis have been conducted in a thorough manner. However, there are a few issues that the authors should address before publication:

Response: We thank the reviewer for the thorough review of the manuscript and the encouraging comments. We have addressed all the comments, and the following are the detailed responses. The revisions made in our manuscript (highlighted by blue color) align with your recommendations.

1) Can the authors present long-time scale dynamics to attest to the reversibility of the transformation observed by the authors?

Response: As requested, we provide and show the scattering intensity lineout for a delay time of 100 ps at an absorbed energy density of 2.55 MJ kg^{-1} in Fig. R2. This is the longest time delay that we acquired during the experiment. The scattering lineout clearly indicates the persistence of the disordered liquid structure at this delay time. It is necessary to mention that the material response under the given strong conditions is not reversible due to the final ablation of thin film samples.

2) the authors have not reported any estimation of the Lindemann limit for Copper, which should be compared with the experimental data

Response: We have now estimated the Lindemann ratio for copper based on the mean square displacement (MSD) model employed in Supplementary Figure 3a of the original manuscript to extract ion temperature from the Debye-Waller analysis.

At the nominal melting temperature of copper ($T_m = 1358$ K), the Day-Shepard model, which was adopted in this work, predicts a relative change of MSD, $\Delta\langle u^2\rangle = 0.0975$ Å^2. Adding the room temperature MSD of 0.0187 Å^2 gives an absolute MSD of $\langle u^2\rangle = 0.1162$ Å^2. Using the nearest neighbor distance $d = 2.56$ Å for copper and the Lindemann relation $\langle u^2\rangle = \delta^2 d^2$, we obtain $\delta \simeq 0.13$ as the critical ratio of root-mean-square amplitude of thermal vibration to nearest-neighbor distance at melting. For comparison, the other two MSD models shown in Supplementary Figure 3a yield $\delta \simeq 0.11$.

Reported Lindemann ratios for fcc metals span a range depending on how the MSD is evaluated. Gilvarry reported values around 0.11 for fcc crystals [J. J. Gilvarry, Phys. Rev. 103, 1700 (1956)], and values as low as 0.08 for copper when the ratio was inferred from elastic properties by extrapolating the bulk modulus under the assumption of a temperature-independent Poisson ratio, which tends to underestimate the ratio as elastic constants evolve near melting.

Because the present estimate is derived directly from experimentally determined MSD and includes anharmonic contributions, the resulting value of ~ 0.13 is slightly higher than harmonic or elastic-extrapolation estimates but remains within the commonly reported range of $0.10 - 0.15$ for crystalline materials [H. Löwen, Phys. Rep. 237, 249 (1994)].

Following the suggestion, we have turned the above-mentioned information to a new subsection titled "Estimate of Lindemann ratio for copper melting" in the Methods section of the revised manuscript.

3) It is not clear how the authors can rule out possible strain effects in taking into account the lag time for the FWHM of the diffraction peak (3 ps). Can the authors further comment on this issue?

Response: Yes, we would like to clarify the concern here. For MeV electron scattering in a normal incidence transmission geometry, one of the key features is that it is only sensitive to in-plane lattice motion because of its near zero Bragg angle. For instance, the Bragg angle for 3.2 MeV electrons reflecting off the (220) lattice plane of copper is $\theta_B = 0.076°$. Under our experimental conditions, only uniaxial lattice expansion along the out-of-plane direction is expected to occur in response to laser heating, see Fig.4a and MD simulations in Ref. 42 [Lin et al. PRB 73, 184113 (2006)]. We therefore do not anticipate to see any strain effects associated with the lattice expansion in our data. This is clearly seen by the time independence of (220) center position over the heating process, as indicated in Fig. R3 that shows the result for the case of 2.55 MJ kg^{-1}.

4) It is not clear the physical argument behind the analysis in Figure 3 and why the peak (111) was chosen. Also, no error bars are included. Can the authors further comment on this issue?

Response: we chose (111) to normalize higher-order diffraction peaks for two main reasons: 1) it helps minimizing the effect of shot-to-shot fluctuation in the electron bunch charge, and 2) it has the most strong scattering signal because of the Q^2 dependence of Debye-Waller effect.

Following the suggestion, we added the error bars for Figure 3b and Extended Data Figure 1b in the revised manuscript.

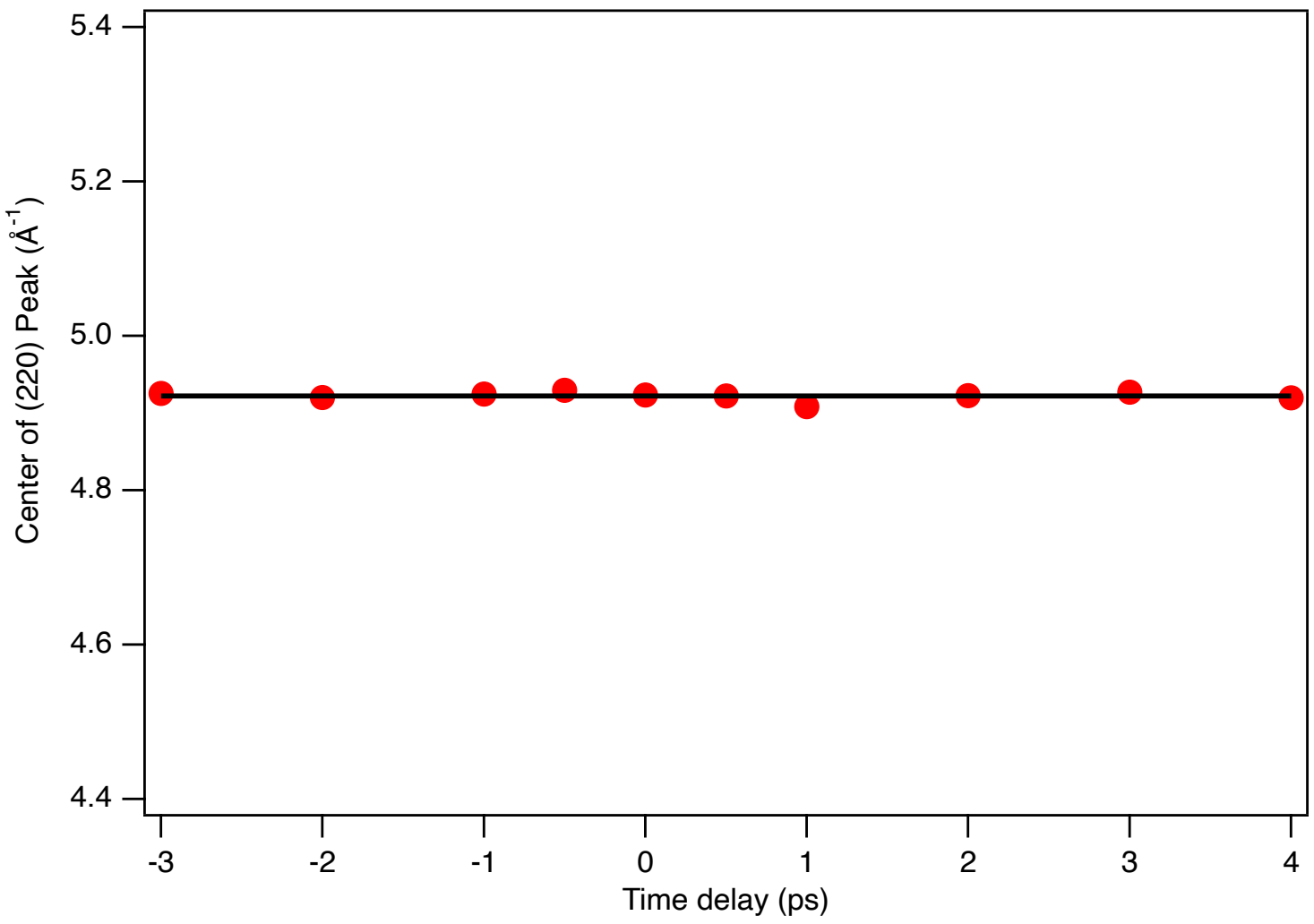

Figure R3: The center position of (220) solid diffraction peak as a function of time delay. The red solid dots are experimental data, while the black solid line is their average value. The energy density in this case is 2.55 MJ kg^{-1}.

Overall, I believe that the manuscript might potentially be published provided that the authors gives precise answers to my previous comments.

Response: We thank the reviewer again for the thorough review of the manuscript and hope that our responses have addressed the comments clearly and satisfactorily, and that the revisions have improved the manuscript.
